# Relationship Between Mitochondrial Structure and Bioenergetics in *Pseudoxanthoma elasticum* Dermal Fibroblasts

**DOI:** 10.3389/fcell.2020.610266

**Published:** 2020-12-17

**Authors:** Francesco Demetrio Lofaro, Federica Boraldi, Maria Garcia-Fernandez, Lara Estrella, Pedro Valdivielso, Daniela Quaglino

**Affiliations:** ^1^Department of Life Sciences, University of Modena and Reggio Emilia, Modena, Italy; ^2^Department of Human Physiology, Biomedical Research Institute of Málaga, University of Malaga, Málaga, Spain; ^3^Department of Medicine and Dermatology, Instituto de Investigación Biomédica de Málaga, University of Malaga, Málaga, Spain; ^4^Internal Medicine Unit, Hospital Virgen de la Victoria, Málaga, Spain

**Keywords:** OCR, ultrastructure, morphology, proteome, mitochondria, fibroblast, PXE

## Abstract

*Pseudoxanthoma elasticum* (PXE) is a genetic disease considered as a paradigm of ectopic mineralization disorders, being characterized by multisystem clinical manifestations due to progressive calcification of skin, eyes, and the cardiovascular system, resembling an age-related phenotype. Although fibroblasts do not express the pathogenic *ABCC6* gene, nevertheless these cells are still under investigation because they regulate connective tissue homeostasis, generating the “arena” where cells and extracellular matrix components can promote pathologic calcification and where activation of pro-osteogenic factors can be associated to pathways involving mitochondrial metabolism. The aim of the present study was to integrate structural and bioenergenetic features to deeply investigate mitochondria from control and from PXE fibroblasts cultured in standard conditions and to explore the role of mitochondria in the development of the PXE fibroblasts’ pathologic phenotype. Proteomic, biochemical, and morphological data provide new evidence that in basal culture conditions (1) the protein profile of PXE mitochondria reveals a number of differentially expressed proteins, suggesting changes in redox balance, oxidative phosphorylation, and calcium homeostasis in addition to modified structure and organization, (2) measure of oxygen consumption indicates that the PXE mitochondria have a low ability to cope with a sudden increased need for ATP *via* oxidative phosphorylation, (3) mitochondrial membranes are highly polarized in PXE fibroblasts, and this condition contributes to increased reactive oxygen species levels, (4) ultrastructural alterations in PXE mitochondria are associated with functional changes, and (5) PXE fibroblasts exhibit a more abundant, branched, and interconnected mitochondrial network compared to control cells, indicating that fusion prevail over fission events. In summary, the present study demonstrates that mitochondria are modified in PXE fibroblasts. Since mitochondria are key players in the development of the aging process, fibroblasts cultured from aged individuals or aged *in vitro* are more prone to calcify, and in PXE, calcified tissues remind features of premature aging syndromes; it can be hypothesized that mitochondria represent a common link contributing to the development of ectopic calcification in aging and in diseases. Therefore, ameliorating mitochondrial functions and cell metabolism could open new strategies to positively regulate a number of signaling pathways associated to pathologic calcification.

## Introduction

*Pseudoxanthoma elasticum* (PXE) is a genetic disease considered as a paradigm of ectopic mineralization disorders. The clinical phenotype reminds of premature aging syndrome (Tiemann et al., 2020), being characterized by multisystem clinical manifestations due to progressive calcification of skin, eyes, and the cardiovascular system (Quaglino et al., 2011; Murro et al., 2018; Li Q. et al., 2019; Murro et al., 2019). The patho-mechanisms of PXE, as well as the complex pathways responsible for hydroxyapatite deposition in soft connective tissues, are still under investigation (Quaglino et al., 2011, 2020).

Although a number of genes may be involved (Li Q. et al., 2009; Omarjee et al., 2019; Boraldi et al., 2020a), PXE is mostly associated with ATP-binding cassette sub-family C member 6 (*ABCC6*) mutations (Le Saux et al., 2000), even though *ABCC6* is not expressed at the protein level in cells of tissues undergoing calcification (Matsuzaki et al., 2005). It was therefore proposed to consider PXE as a metabolic disease (Jiang et al., 2009), leaving questionable the role of mesenchymal cells within this context. Intriguingly, fibroblasts from PXE patients (Ronchetti et al., 2013) are characterized by an altered protein profile (Boraldi et al., 2009), increased oxidative stress (Pasquali-Ronchetti et al., 2006; Garcia-Fernandez et al., 2008), enhanced proteolytic potential (Quaglino et al., 2005), and a higher propensity to calcify (Boraldi et al., 2014a). Interestingly, it was observed that, even before the development of calcification, fibroblasts isolated from the skin of *Abcc6*-/- mice exhibit altered features (Boraldi et al., 2014b). Therefore, a theory was proposed that mesenchymal cells can actively contribute to the formation/accumulation of mineral precipitates, although it cannot be excluded that other phenotypic changes are further generated by the mineralized environment.

The factor/s triggering the initial core of hydroxyapatite deposition still has/have to be identified. Degraded elastin fragments, *per se*, can expose multiple charged sites, favoring interactions with calcium and phosphate (Boraldi et al., 2020b). In addition, pro-osteogenic stimuli driven by necrotic cell debris, inflammatory cytokines, or unbalanced calcium-phosphate homeostasis can modify the transcriptional program and promote the differentiation of soft connective tissue mesenchymal cells into osteogenic cells releasing bone-related molecules (Mathieu and Boulanger, 2014).

Previous *in vitro* experiments demonstrated that, in fibroblasts, redox balance and extracellular matrix homeostasis were affected by aging and that, similarly to PXE fibroblasts (Boraldi et al., 2013), aged cells were more susceptible to pro-osteogenic signals (Boraldi et al., 2010, 2015). Since the aging process has been associated to disrupted mitochondrial function (Miquel and Fleming, 1984; Bornstein et al., 2020), it can be suggested that mitochondria can also contribute to the pathologic phenotype of PXE fibroblasts.

To broaden the possible role of mitochondria in the calcification process, recent evidence by exome sequencing revealed that, in patients affected by pathologic mineralization, altered extracellular matrix homeostasis and activation of pro-osteogenic factors can be associated to pathways involving mitochondria metabolism (Boraldi et al., 2019; Lee et al., 2020).

The aim of the present study was to integrate structural and bioenergenetic features to deeply investigate mitochondria from control and from PXE fibroblasts cultured in standard conditions and to explore the role of mitochondria in the development of the PXE fibroblasts’ pathologic phenotype.

## Materials and Methods

### Cell Cultures

Human dermal fibroblasts were selected within the cryo-stored cell collection of the laboratory, paying special attention to use cells at similar passages, from the same site of biopsy and from adults in the absence of comorbidities. In particular, fibroblasts were isolated from biopsies taken from the arms of three female patients affected by PXE (45 ± 14) and from three healthy (Ctr) females (46 ± 12) after informed consent in accordance with the World Medical Association’s Declaration of Helsinki. All subjects were of Italian origin and did not exhibit any sign of disorders such as diabetes, hypertension, lipid metabolism abnormalities, and kidney or liver diseases. The PXE patients were diagnosed by clinical (dermal and ocular involvement) and biomolecular findings (known *ABCC6* pathogenic mutations). All patients exhibited a similar clinical phenotype characterized by lax and redundant skin and by ocular bleeding and choroidal neovascularization. Patient #1 was compound heterozygous for a stop codon mutation (c.3421C > T; p.Arg1141^∗^) and for a large deletion in exon 23_29 (c.2996_4208del p.Ala999_Ser1403del); patient #2 was compound heterozygous for two stop codon mutations (c.1552C > T, p.Arg518^∗^ and c.3088c > T, p.Arg1030^∗^); and patient #3 was compound heterozygous for a missense mutation (c.4198G > A, p.Glu1400Lys) and for a deletion in exon 23_29 (c.2996_4208del p.Ala999_Ser1403del) (Quaglino et al., 2011, 2020). The fibroblasts were cultured, as already described with some modification (Quaglino et al., 2000), using Dulbecco’s Modified Eagle Medium (DMEM; Gibco, Grand Island, NY, United States) supplemented with 10% fetal bovine serum and non-essential amino acids 1 × (Gibco), but without penicillin and streptomycin because antibiotics may interfere with mitochondrial respiration and reactive oxygen species (ROS) production. Fibroblasts were used at sub-confluence between the 6th and 9th passages, and all experiments were done on each cell line at least three times and in triplicate to account for technical variability, with the only exception for the mitochondria proteome (see below). Furthermore and most importantly, the cells were analyzed, when technically feasible, adherent to their substrate or treated in order to limit stresses due to enzymatic cell detachment that causes changes in cell shape and consequently in mitochondria structure, distribution, and function.

### Mitochondria Isolation and Protein Extraction

Since primary cell cultures take more time to grow than continuous cell lines and the mitochondrial number/fibroblast is relatively low compared to other cells (i.e., hepatocytes), in order to have a sufficient number of mitochondria for proteome analysis, mitochondria were isolated from 6 × 10^7^ cells pooled together from three control or three PXE cell lines according to the Panov protocol (Panov, 2015).

The two cell pools were manually lysed with a glass Dounce homogenizer using a hypotonic swelling buffer (100 mM sucrose, 10 mM MOPS, 1 mM EGTA-KOH, 0.1% BSA, pH 7.2). Then, a hypertonic solution (1.25 M sucrose, 10 mM MOPS, pH 7.2) was added, and the samples were centrifuged (1,000 × *g* for 5 min at 4°C). Supernatants were collected and further centrifuged (9,000 × *g* for 10 min at 4°C) to isolate the crude mitochondria fraction that was then suspended in isolation buffer (75 mM mannitol, 225 mM sucrose, 10 mM MOPS, pH 7.2) and layered on a sucrose gradient (1.5 M/1.0 M sucrose in 10 mM Hepes-KOH, 1 mM EDTA-KOH, 0.1% BSA, pH 7.2). The samples were ultra-centrifuged at 96,000 × *g*, for 2 h at 4°C in a Beckman Ultracentrifuge with a SW40Ti rotor. After ultracentrifugation, a whitish band appeared at the gradient interface, allowing us to collect mitochondria. To perform shotgun proteomics, the isolated mitochondria were sonicated, and proteins were recovered using methanol/chloroform precipitation in order to eliminate sucrose. The proteins were resuspended in 50 mM NH_4_HCO_3_ (Merck, Milano, Italy), and protein content was quantified by Bradford method (Bradford, 1976). The proteins (50 μg) were reduced in the presence of 5 mM 1–4 dithiothreitol (Merck), alkylated with 15 mM iodoacetamide (Merck) in the dark, and digested overnight at 37°C with trypsin (Promega, Madison, WI, United States) at an enzyme-to-protein ratio of 1:50 (w/w). Three independent experiments were performed in duplicate on each cell pool.

### Liquid Chromatography and Mass Spectrometry Analysis

Mitochondrial peptides were analyzed using a UHPLC ultimate 3000 system coupled online to a Q Exactive Hybrid Quadrupole-Orbitrap Mass Spectrometer (Thermo Fisher Scientific, Waltham, MA, United States). A reverse-phase C18 column (50 mm × 2.1 μm ID, 1.8 μm, Zorbax) was used to perform peptide chromatographic separation. Elution was conducted using a mobile phase A of 0.1% formic acid in ultrapure water and a mobile phase B of 0.1% formic acid in acetonitrile. For separation, a linear binary gradient was applied: 2–3% B in 5 min to 28% B in the next 59 min and then 90% B in 7 min. The column was maintained at 30°C, and the flow rate employed was 0.3 ml/min. The precursor ion detection was done in a m/z range from 200 to 2,000; the acquisition range for fragment ions was m/z from 200 to 2,000. Data acquisition was controlled by Xcalibur 2.0.7 Software (Thermo Fisher Scientific).

### Analysis of MS/MS Data and Bioinformatics

Raw ms/ms data were converted by msConvert ProteoWizard (v.3.0.19239) in MGF file using default settings and uploaded to MASCOT server (v.2.4.0) for MS/MS Ion Search.

Search was performed using Uniprot (2018_05) restricted to *Homo sapiens* (ID 9606). Furthermore, the parameters for identification included the following: (i) trypsin as enzyme with one as maximum missed cleavage, (ii) mass error tolerances for precursor and fragment ions set to 10 ppm and 0.02 Da, respectively, (iii) peptide charge (2+, 3+, 4+), (iv) protein mass no restriction, and (v) carbamidomethyl cysteine was set as fixed modification, while deamination of asparagine and glutamine and oxidation of methionine were considered as dynamic modification. Label-free quantitation using spectral counting emPAI was performed by MASCOT. Only confident peptide identified with a false discovery rate ≤1% and protein with at least one unique peptide were exported. Proteins appearing in at least two biological replicates of one group (control or PXE) but were never detected in the other group (PXE or control) were considered as unique. Proteins were classified as mitochondrial or predicted to be mitochondrial using MitoMiner (v.4.0) (Smith and Robinson, 2009). Lastly, protein–protein interaction network was built using STRING (v.1.5.1) implemented in Cytoscape (v. 3.8.0) with a confidence score of 0.7 and 0 additional interactors (Shannon et al., 2003).

### Oxygen Consumption Rate and Mito-Stress Test

Oxygen consumption rate was measured using the Seahorse XFe24 Analyzer (Agilent Tecnologies, Santa Clara, CA, United States). Briefly, at 24 h before the assay, each cell line (4 × 10^4^ cells/well) was seeded in four wells/case of Seahorse plate and incubated at 37°C with 5% CO_2_. On the following day, the cells were equilibrated with pre-warmed Seahorse XF assay medium (DMEM with 10 mM glucose, 2 mM L-glutamine, and 1 mM sodium pyruvate without NaHCO_3_, pH 7.4) at 37°C for 1 h. The oxygen consumption rates (OCR) of the cellular monolayer were measured before (basal level) and after the sequential injection of 1 μM oligomycin (blocks proton translocation through complex F0/F1, inhibiting ATP production and reducing OCR), 0.5 μM rotenone and 0.5 μM antimycin A (shut down the mitochondrial respiration inhibiting complex I and III, respectively, and reducing OCR to a minimal value), and 1 μM carbonyl cyanide-4-(trifluoromethoxy)phenylhydrazone (FCCP) (a potent uncoupler of mitochondrial oxidative phosphorylation). In particular, injection of FCCP, disrupting ATP synthesis by transporting protons across the mitochondrial inner membrane and interfering with the proton gradient, was used to measure maximum OCR that depends on the electron transport capacity and substrate delivery. The cellular bioenergetic parameters determined were as follows: basal OCR was obtained by subtracting the respiration rate after the addition of antimycin A and rotenone (non-mitochondrial OCR) to the baseline respiration, ATP-linked OCR was derived from the difference between basal OCR and respiration following oligomycin addition, proton leak was the difference between respiration following oligomycin addition and non-mitochondrial OCR, and maximal OCR was determined by subtracting the non-mitochondrial OCR to the OCR induced by FCCP. Lastly, spare capacity was calculated by the difference between maximal OCR and basal OCR. Three measurements were taken after each injection. The experiments were performed in quadruplicate on each cell line. Data were normalized to the protein content of each well (Roy-Choudhury and Daadi, 2019) by the Bradford method (Bradford, 1976).

### Mitochondrial Superoxide Detection

MitoSOX Red is a novel fluorescent dye that specifically targets the mitochondria and is used to detect mitochondrial superoxide. This technique was applied to control and PXE cells. The adhered cells were treated for 20 min at 37°C with 2.5 μM MitoSOX (Thermo Fisher Scientific) in complete medium. Then, the cells were detached, washed, and resuspended in 250 μl of PBS and analyzed with Attune Nxt (Thermo Fisher Scientific) flow cytometer. MitoSOX Red was excited by laser at 488 nm, and emitted fluorescence was detected with a bandpass filter of 585/42 nm. At least 10,000 total events were acquired for each sample. An unstained sample for each cell line was acquired to set the level of autofluorescence.

### Confocal Microscopy

#### MitoView Staining in Live Cells

To visualize and to evaluate the cellular mitochondrial network, cells were grown on a four-well glass plate at a density of 3 × 10^3^ cells/well in 500 μl of culture medium for 24 h. Fibroblasts at sub-confluence were then incubated with 50 nM MitoView (Biotium, Hayward, CA, United States) and 0.1 μg/ml Hoechst 33342 at 37°C for 15 min and were kept at 37°C in an Okolab incubation chamber. A positive control of the reaction was represented by incubation with 100 μM mitochondrial uncoupler carbonyl cyanide m-chlorophenyl hydrazone (CCCP). Images were taken with ×63 Plan-Apo oil immersion objective mounted on a Leica SP8 confocal microscope equipped with a white light laser. The acquired images (10 photos for each biological and technical replicate) were analyzed using Mitochondria Analyzer (v.2.0.2^1^), a mitochondrial morphology plugin developed in ImageJ software (v.1.53c) (Schindelin et al., 2012; Rueden et al., 2017). Each single mitochondrion was analyzed for morphological characteristics such as area, perimeter, circularity, and major/minor axis. On the basis of these parameters, the form factor [perimeter^2^/(4π × area)] and the aspect ratio (ratio between the major and minor axes of the ellipse equivalent to the object) were calculated, representing the degree of mitochondrial branching and the length of mitochondria, respectively (Krebiehl et al., 2010). Morphological parameters include (1) form factor, a shape descriptor where a circular object has a value close to 1, while a value >1 indicates other shapes, and (2) aspect ratio, morphological parameters ranging from 0 (circular shape) to 1 (elongated shape). Network parameters include branch length per mitochondrion, a ratio between the mean of branch length and mitochondria in each image, branch junction per mitochondria, and number of junctions per mitochondria in each image. Junctions are points of contact of two or more branches.

#### Mitochondrial Membrane Potential (Δψ_m_) in Live Cells

To evaluate Δψ_m_, we used the cyanine dye JC-1 (5,5′,6,6′-tetrachloro-1,1′,3,3′-tetraethylbenzimidazolocarbo-cyanine iodide) (Thermo Fisher Scientific), and stained cells were observed by confocal microscopy. JC-1 has the ability to accumulate within polarized mitochondria, forming red fluorescence-emitting aggregates, whereas it cannot aggregate in the mitochondrial matrix (green-emitting dye monomers) when the Δψ_m_ is low. It is to be noted that the red/green fluorescence ratio depends on membrane potential and not on mitochondrial shape, density, or size.

In particular, fibroblasts were seeded in a four-well glass plate at a density of 3 × 10^3^ cells/well in 500 μl culture medium in a 5% CO_2_ incubator overnight at 37°C. After 1 day of seeding, the cells were incubated with 3.0 μM JC-1 in DMEM without phenol red in a 5% CO_2_ incubator at 37°C for 20 min. As a positive control, cells were treated with 100 μM CCCP to depolarize the mitochondria.

The cells were kept at 37°C in an Okolab incubation chamber, and images were acquired using a ×63 oil immersion objective with a confocal microscope. JC-1 dye was excited with a 405 laser (Perelman et al., 2012), and image acquisition was performed using a 500–551-nm detection bandwidth (green signal) and a 560–651-nm bandwidth (red signal). The confocal images were analyzed using the Olympus ScanR software. Briefly, mitochondria were segmented using the Edge module over the red channel of the JC-1 signal. The mean intensity of the red and of the green signals were determined for each mitochondrion, and the ratio of the red over the green signal was calculated.

#### Calcium Imaging in Live Cells

A total of 1.5 × 10^4^ cells were seeded onto four-chamber slides and cultured for 24 h with complete medium. On the following day, the cell monolayer was washed twice with complete medium without phenol red and incubated with 2.5 μM Rhod-2 AM (CliniSciences, Roma, Italy) for 30 min at 37°C with 5% CO_2_. The cells were then washed three times with DMEM without phenol red, kept at 37°C in an Okolab incubation chamber, and observed with a confocal microscope. A positive control of the reaction was represented by incubation with 100 μM CCCP. Images were taken of the cellular monolayer in each well. As for JC-1 image analysis, mitochondria were segmented using the Edge module, implemented in Olympus ScanR software, over the red channel of the Rhod-2-AM signal, allowing us to evaluate as much as 30,000 mitochondria for each cell line. The mean fluorescence intensity of the red signal was therefore determined for each mitochondrion.

### Transmission Electron Microscopy

Fibroblasts were physically scraped from the plastic substrate, centrifuged, and fixed in 2.5% glutaraldehyde (Electron Microscopy Sciences, Hatfield, PA, United States) dissolved in 0.1 M cacodylate buffer at pH 7.4 for 12 h. Post-fixation was performed for 60 min in 1% osmium tetroxide using the same buffer. Samples were dehydrated with increasing ethanol concentration up to 100% and embedded in Epon 812 (Electron Microscopy Sciences). Ultrathin sections (60 nm) were cut and mounted on 150 mesh copper grids (Electron Microscopy Sciences). Unstained samples were observed with Talos F200S G2 transmission electron microscope (Thermo Fisher Scientific).

### Statistical Analyses

Data were analyzed using GraphPad Prism 8.0 (San Diego, CA, United States), and the results are reported as mean ± SEM. The statistical significance of the difference between two experimental groups was determined by two-tailed unpaired Student’s *t*-test. Values with *p* < 0.05 were considered as statistically significant.

## Results and Discussion

### Shotgun Proteomics Analysis Reveals Changes in Mitochondrial Protein Profile

To provide a global overview of protein composition, the proteomic profile was analyzed in mitochondria from PXE and from control fibroblasts.

After taking into consideration only proteins identified in at least two out of three biological replicates in PXE and in control cells, a total number of 397 different proteins (Supplementary Table 1) were assigned to a specific function according to the MitoMiner (v.4.0) database (Smith and Robinson, 2009), a large-scale proteomic dataset including the MitoCarta Inventory and the Integrated Mitochondrial Protein Index, which have been determined by experimental and bioinformatic predictions, respectively. A high number of identified proteins (270 out of 397; 68) were classified as known or predicted to be mitochondrial proteins. The mitochondrial enrichment procedure applied to increase mitochondrial protein abundance exhibited an efficiency comparable to that observed in other studies (Alberio et al., 2017; Maffioli et al., 2020). The presence of non-mitochondrial proteins (i.e., endoplasmic reticulum, cytoskeletal proteins) is due to the physical connection between the mitochondria and other subcellular organelles (Dolman et al., 2005; Rowland and Voeltz, 2012; Xia et al., 2019; Giacomello et al., 2020) as already demonstrated in previous studies (Rezaul et al., 2005; Alberio et al., 2017; Chae et al., 2018). It is well known that mitochondria can be in close contact with the endoplasmic reticulum, forming mitochondria-associated membrane and playing a role in lipid biosynthesis, mitochondrial biogenesis/dynamics, and calcium exchange (Csordás et al., 2006). Moreover, the association of mitochondria to cytoskeletal proteins can influence mitochondrial motility, dynamics, and morphology (Rappaport et al., 1998; Knowles et al., 2002; Appaix et al., 2003).

**FIGURE 1:**
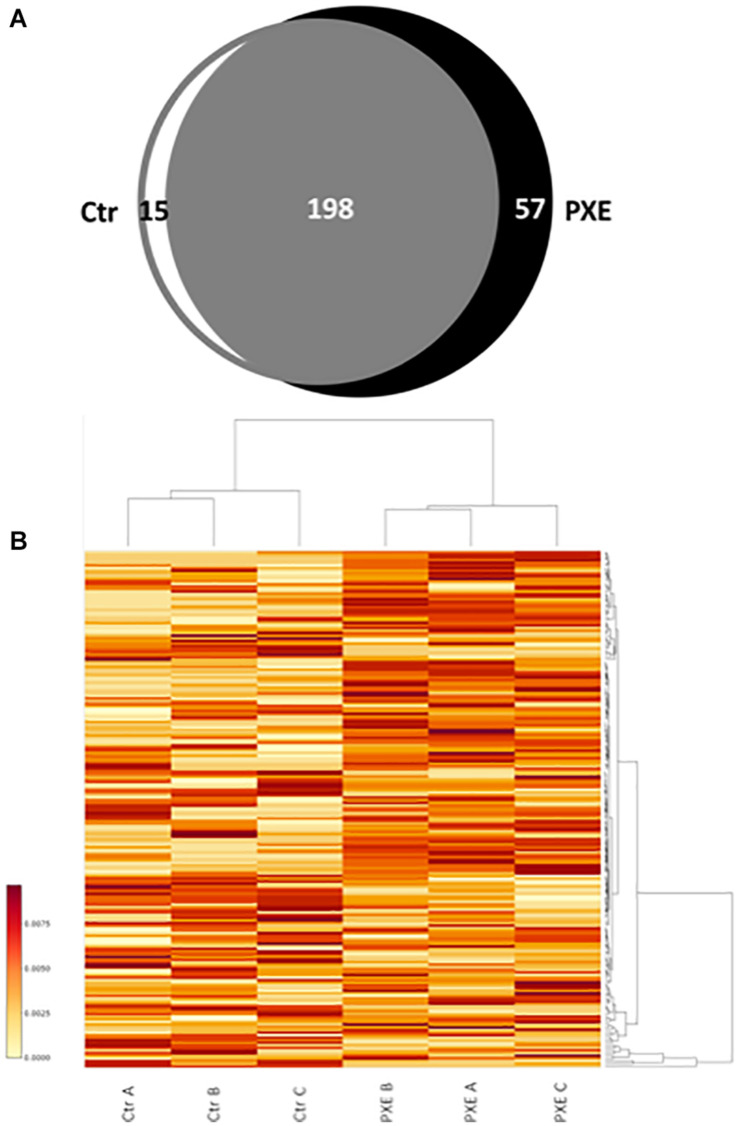
**(A)** Venn diagram of identified proteins referred to as mitochondrial proteins in control (Ctr) and *Pseudoxanthoma elasticum* (PXE) fibroblasts. **(B)** Heat map of relative abundance of differentially expressed mitochondrial proteins in a pool of three Ctr and of three PXE samples. Three independent experiments (A, B, and C biological replicates) were performed in duplicate (technical replicates). The colored bar indicates the expression levels.

A comparison of identified mitochondrial proteins (270) demonstrates that 198 proteins were detected in both control and PXE cells and were considered as “common” (Supplementary Table 2), whereas 15 and 57 proteins were detected only in control or in PXE fibroblasts, respectively, and were therefore considered “unique” as clearly differentially expressed (Taverna et al., 2015; Figure 1A and Supplementary Table 3).

In order to evaluate the differential expression of proteins common to both control and PXE samples, we performed a relative quantification using a MS-based label-free approach, allowing us to compare the protein amount in two or more samples (Bantscheff et al., 2012; Calderón-Celis et al., 2018) based on counting of the MS^2^ spectra from the peptides of a given protein. Significant changes in the expression of common proteins were reported in the heat map (Figure 1B), where proteins correspond to rows and samples to columns.

**FIGURE 2:**
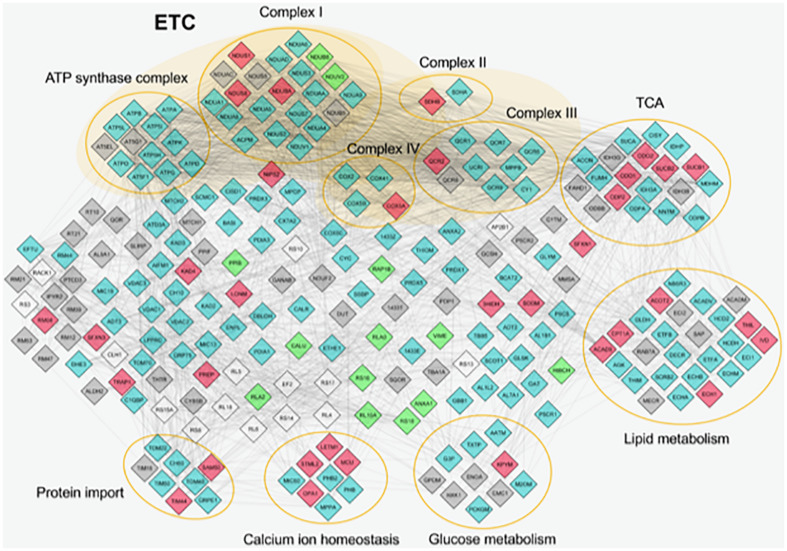
Protein–protein interactions of mitochondrial proteins identified by proteome analysis. The nodes represent individual proteins; the edges (lines) represent all cross-links identified among proteins. The nodes are colored in different ways on the basis of their abundance. Light blue, proteins expressed in similar way between control and *Pseudoxanthoma elasticum* (PXE); red, common proteins more expressed in PXE; green, common proteins more expressed in control; gray, unique proteins in PXE; white, unique proteins in control. Proteins without connections are excluded. ETC, electron transport chain; TCA, tricarboxylic acid cycle.

Furthermore, STRING database and GO enrichment analysis were also applied to all identified proteins to explore protein–protein interactions in order to reveal the involved pathways (Figure 2).

Most of the differentially expressed proteins were related to metabolic enzymes (e.g., pyruvate kinase, acetyl-CoA acetyltransferase, acyl-CoA dehydrogenase, 3-hydroxyisobutyrate dehydrogenase, peptidyl-prolyl cis-trans isomerase B), indicating that mitochondria can contribute to PXE metabolic changes (Kuzaj et al., 2014).

A significantly overexpressed protein linked to energy metabolism is inorganic pyrophosphatase 2 (IPYR2) that, similarly to the cytosolic IPYR, catalyzes the hydrolysis of pyrophosphate to inorganic phosphate (Kennedy et al., 2016). The role of this protein and the possible contribution to hydroxyapatite formation deserve further investigation in future studies.

Moreover, in PXE mitochondria, significant changes have been observed in proteins involved in oxidative stress [i.e., overexpression of superoxide dismutase (Mn) (SODM), tumor necrosis factor receptor-associated protein 1 (TRAP1), and Lon protease homolog (LONM)]. SODM is a mitochondrial enzyme involved in the conversion into H_2_O_2_ of O_2_^⋅–^ formed during mitochondrial respiration. TRAP1 or HSP75 is a member of the HSP90 family controlling a variety of cellular functions such as cell proliferation, differentiation, and survival (Johnson, 2012; Im, 2016). It regulates the metabolic switch between oxidative phosphorylation and aerobic glycolysis, and its overexpression is associated with increased ROS levels (Im, 2016). LONM, a mitochondrial ATP-dependent protease, plays an important role in maintaining mitoproteostasis through selectively recognizing and degrading oxidative damaged proteins, and it is overexpressed by altered redox balance (Gibellini et al., 2014, 2020). In turn, up-regulated LON increases mitochondrial ROS generation and, as a pivotal stress-responsive protein, is involved in the crosstalk among mitochondria, endoplasmic reticulum, and nucleus (Yang et al., 2018). Taken together, the changes in the expression of these proteins indicate an active role of mitochondria in the PXE-altered redox homeostasis.

Altered protein expression was observed in different respiratory complexes. For example, among the subunits of complex I (NADH-ubiquinone oxidoreductase), several proteins were up-regulated (e.g., NDUS1, NDUAC, NDUBA) or down-regulated (e.g., NDUB8, NDUV2) in PXE mitochondria, whereas in complex IV (cytochrome oxidase) COX5A appeared markedly up-regulated in PXE. Therefore, protein alterations in different respiratory complexes can affect the mitochondrial oxidative phosphorylation system (OXPHOS), which could, in turn, induce the further production of ROS.

Among proteins involved in calcium homeostasis, proteomic analysis revealed in PXE mitochondria an up-regulation of stomatin-like protein 2 (STML2), dynamin-like 120-kDa protein (OPA1), and mitochondrial calcium uniporter (MCU). The first is a mitochondrial inner membrane protein involved in mitochondria biogenesis, calcium homeostasis, and mitochondrial membrane organization (Lapatsina et al., 2012). Interestingly, it is involved in the mild stress-induced mitochondria hyperfusion as a pro-survival response, acting in synergy with OPA1, a protein that is required for mitochondrial fusion and which was highly expressed in PXE mitochondria. Cell energy metabolism, ROS production, and calcium homeostasis, besides STML2, are further modulated by the overexpression of MCU, a calcium uniporter protein acting as a primary mediator of Ca^2+^ influx into the mitochondria (Takeuchi et al., 2015) through a process dependent on the membrane potential of the inner mitochondrial membrane (Shah and Ullah, 2020). It is to be noted that MCU allows rapid and massive Ca^2+^ entry at high cytosolic Ca^2+^ concentrations (>10 μM). By contrast, the similarly up-regulated mitochondrial proton/calcium exchanger protein (LETM1), a Ca^2+^/H^+^ antiporter, operates at low cytosolic Ca^2+^ concentrations (<100 nM) and is regulated by the pH gradient generated by the mitochondrial electron transport chain (ETC) (Li Y. et al., 2019). Interestingly, LETM1 seems to be also involved in regulating mitochondria morphology, contributing to its tubular shape and cristae organization (Austin and Nowikovsky, 2019).

Since proteins do not act alone but interact with other polypeptides and macromolecules to modulate specific processes, oxygen consumption, mitochondrial membrane potential (Δψ_m_), ROS and Ca^2+^ levels as well as mitochondrial morphology were investigated to evaluate if the changes detected by proteome analysis had consequences on some biologically relevant processes.

### Mitochondrial Respiratory Capacity in PXE Is Lower Than in Control

**FIGURE 3:**
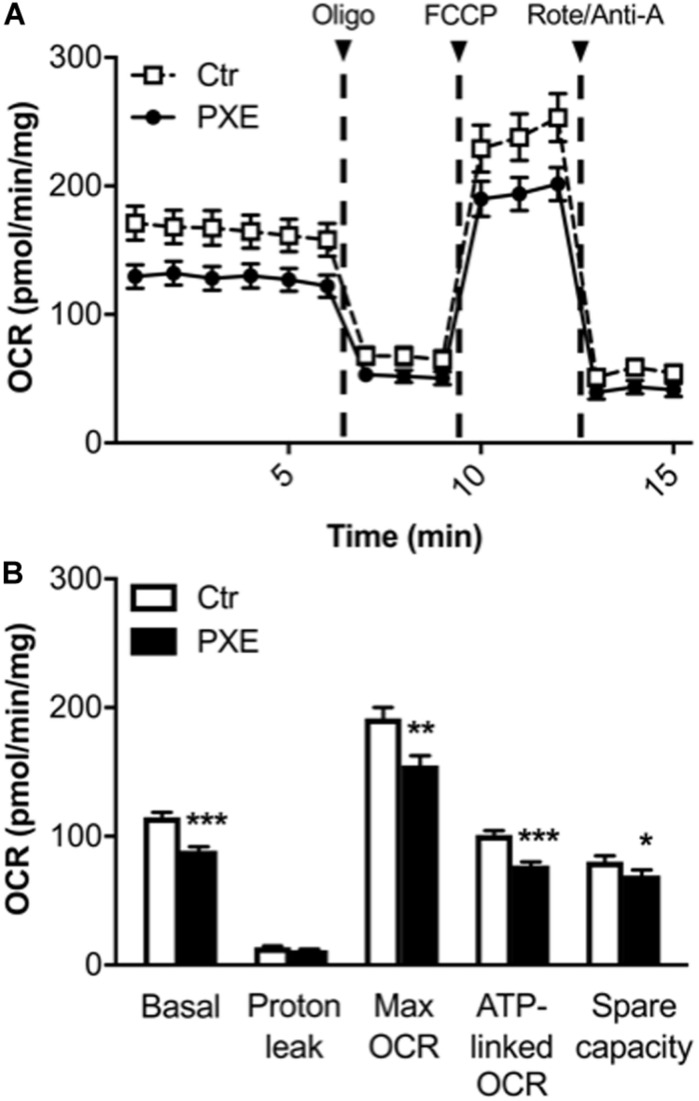
**(A)** Results from the Mito Stress Test comparing control (Ctr) and *Pseudoxanthoma elasticum* (PXE) fibroblasts. To evaluate mitochondrial function, cells were injected with oligomycin (oligo), carbonyl cyanide-4-(trifluoromethoxy)phenylhydrazone, and rotenone/antimycin A. **(B)** Quantification of the basal oxygen consumption rate (OCR), proton leak, maximal OCR, ATP-linked OCR, spare capacity in Ctr and PXE mitochondria (see section “Materials and Methods”). Values are expressed as mean ± SEM of three independent experiments conducted in quadruplicate on each cell line. *p < 0.05; **p < 0.01; ***p-value < 0.001 PXE *vs* Ctr.

A mitochondrial (mito) stress test was performed using the Seahorse analyzer to assay, in a non-invasive and in a real-time manner, oxygen consumption and ATP-linked OCR through the OXPHOS. OCR measurement allows estimating the changes of different parameters related to mitochondrial respiratory function after the sequential injection of specific drugs (see section “Materials and Methods”) (Figure 3). PXE fibroblasts had basal respiratory values and ATP-linked OCR lower than those of control cells, whereas the OCR required for mitochondrial functions other than ATP synthesis (calculated from the difference between the OCR after oligomycin addition and the residual OCR) was similar in both cell lines. A significant decrease of the maximum respiration rate was observed in pathological cells in comparison to control fibroblasts, indicating a reduced substrate availability and/or functional capacity of the ETC. The spare respiratory capacity, an important measure of the ability to respond to stress or to increased workload (Brand and Nicholls, 2011; Hill et al., 2012), was significantly lower in PXE fibroblasts compared to control cells, indicating that PXE cells had a low ability to cope with a sudden increased need for ATP *via* oxidative phosphorylation. If the spare respiratory capacity of the cells is not enough to provide the required ATP, the affected cells may undergo premature senescence or cell death (Desler et al., 2012). Moreover, mitochondrial dysfunction causes the overproduction of ROS, mitochondrial DNA damage, aberrant mitochondrial dynamics, and disturbed calcium homeostasis.

In addition, a decrease in both basal OCR and spare capacity without a significant increase of proton leak in PXE mitochondria is associated with reduced mitochondrial function (Armstrong et al., 2018).

### PXE Mitochondria Are Hyperpolarized and Exhibit Increased ROS

The ability of cells to keep stable levels of intracellular ATP and Δψ_m_ is fundamental for normal cell functioning (Zorova et al., 2018); therefore, differences of these parameters, due to physiological activity, metabolic requirements, or environmental changes, may have consequences on cell viability and/or on disease occurrence.

**FIGURE 4:**
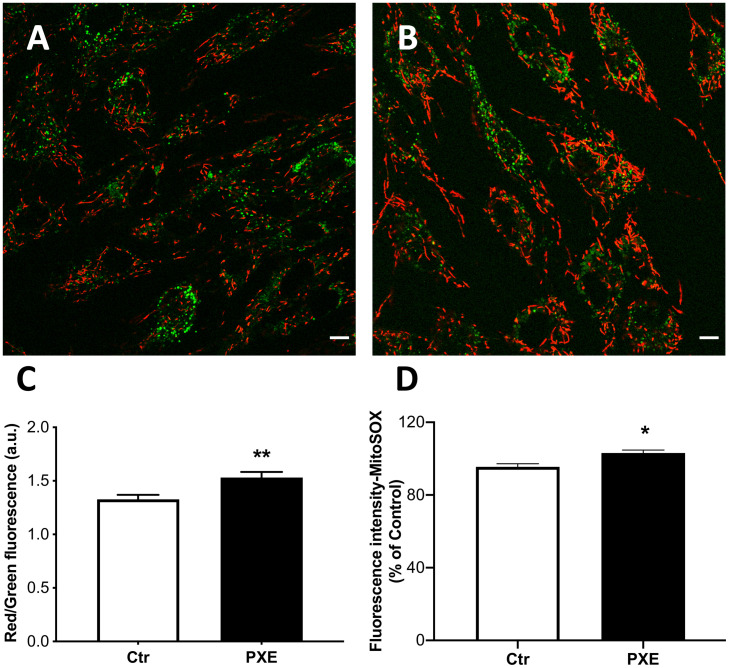
Representative images of mitochondrial membrane potential evaluated in mitochondria from control (Ctr) **(A)** and *Pseudoxanthoma elasticum* (PXE) **(B)** fibroblasts stained with JC-1 and observed by confocal microscopy in a live-imaging mode. The mitochondria in PXE cells were predominantly in red form, indicating a higher membrane potential than those present in control fibroblasts. Bar = 10 μm. **(C)** Histogram showing the ratio of red to green fluorescence intensity measured on control and PXE fibroblasts. **(D)** Mitochondrial O_2_^⋅–^ levels detected in Ctr and in PXE fibroblasts by MitoSOX-based flow cytometry. Data are expressed as mean ± SEM of three independent experiments conducted in triplicate on each cell line. *p < 0.05; **p < 0.01PXE *vs* Ctr.

The cyanine dye JC-1 was used to evaluate, by ratiometric imaging approaches, the Δψ_m_ in control and pathologic fibroblasts (Figures 4A,B). The red/green fluorescence ratio obtained from control (*n* = 90) and PXE (*n* = 90) fibroblasts in monolayer indicated significantly higher Δψ_m_ values in pathologic compared to control cells (Figure 4C). Furthermore, JC-1, on the basis of red fluorescence intensity, can be used also as a quantitative measure (Cossarizza and Salvioli, 1998). Therefore, a segmentation strategy was applied and integrated in the ScanR software to analyze the red signal of approximately 41,000 mitochondria/condition. Data confirm that the red fluorescence from JC-1 aggregates was higher in PXE than in control cells (Ctr = 1,271 ± 2.61 and PXE = 1,310 ± 2.72; ***p < 0.001), clearly indicating high Δψ_m_. CCCP, an uncoupling ionophore used as a positive control, led to massive mitochondrial depolarization in both cell lines (Supplementary Figure 1).

The increase of Δψ_m_ is linked to the extrusion of H^+^ ions from the mitochondrial matrix, and consequently, the cytochromes within ETC, becoming more reduced, favor ROS generation.

Mitochondria were tested for the production of O_2_^⋅–^. The MitoSOX probe was used since it is able to rapidly accumulate into the mitochondria due to its positive charge and yields a fluorescent signal being rapidly oxidized by O_2_^⋅–^, but not by other ROS or reactive nitrogen species. As expected, in basal culture conditions, the intra-mitochondrial levels of O_2_^⋅–^ were very low; however, in PXE, there was a small, but significant, increase of fluorescence compared to control fibroblasts (Figure 4D). These data underline the role of mitochondria in contributing to the sub-chronic oxidative stress condition observed in PXE both *in vitro* and *in vivo* (Pasquali-Ronchetti et al., 2006; Garcia-Fernandez et al., 2008; Boraldi et al., 2014b).

### Low Free Calcium in PXE Mitochondria

**FIGURE 5:**
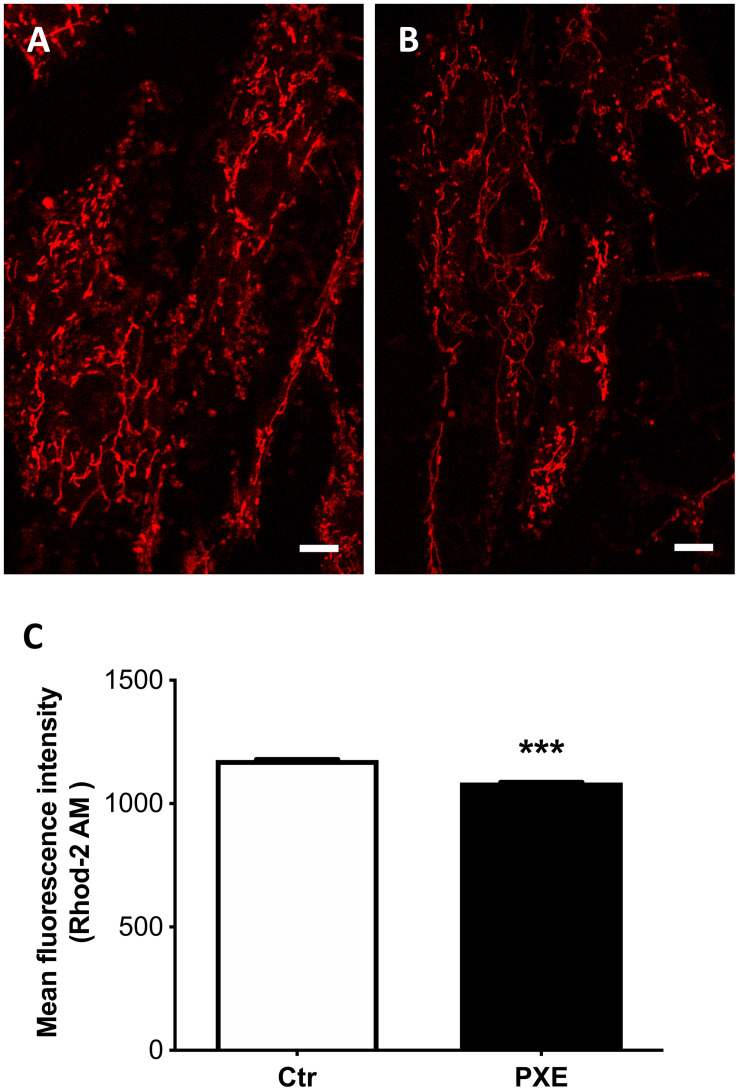
Representative images of mitochondrial free calcium in control (Ctr) **(A)** and in *Pseudoxanthoma elasticum* (PXE) fibroblasts **(B)**. Cells were loaded with Rhod-2 AM to determine mitochondrial calcium levels and observed by confocal microscopy in a live-imaging mode. Bar = 10 μm. **(C)** Quantification of mitochondrial free calcium levels expressed as mean fluorescence intensity in control and in PXE. Data are represented as mean ± SEM of three independent experiments conducted in triplicate on each cell line. ***p < 0.001 PXE *vs* Ctr.

Besides the role of damaging and stressor agents, ROS can regulate both physiological and pathophysiological processes frequently overlapping calcium (Ca^2+^)-mediated signaling pathways controlling membrane potential and mitochondrial ATP production (Madreiter-Sokolowski et al., 2020). Free mitochondrial calcium was evaluated by loading control and PXE fibroblasts (Figures 5A,B) with Rhod-2 AM. The PXE mitochondria were characterized by lower amounts of free calcium compared to controls (Figure 5C). It has been demonstrated that mitochondrial calcium regulates pyruvate dehydrogenase (Denton et al., 1972), tricarboxylic acid cycle enzymes, complex III and IV, and ATP synthase (Tarasov et al., 2012), consistent with the decreased OCR observed in PXE mitochondria.

### Altered PXE Mitochondrial Morphology

**FIGURE 6:**
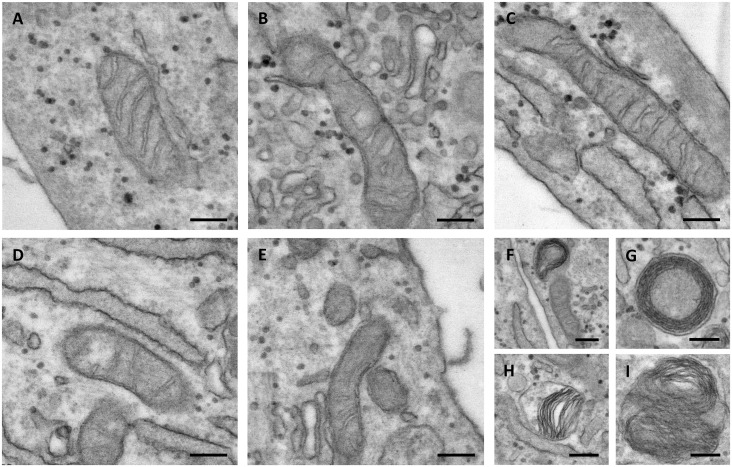
Representative electron microscopy images of mitochondria in control **(A–C)** and in *Pseudoxanthoma elasticum* fibroblasts **(D–I)**. In pathologic cells, the mitochondria can be observed as exhibiting partial loss of cristae **(D,E)**, concentric cristae compartments (“onion-like”) **(F,G)**, warped cristae located on one side of the mitochondria **(H)**, and multiple overlaid layers of outer and inner membranes generating two separate compartments **(I)**. Bar = 200 nm.

Mitochondria are dynamic organelles able to change their shape as well as the size and the number of cristae depending on the energy demand or on other physiological requirements. It is known that, in the mitochondria, form and function are strictly connected; therefore, we have analyzed the ultrastructure of mitochondria from cultured control and PXE fibroblasts (Figure 6).

The control mitochondria were characterized by a typical elongated shape and by abundant cristae. The presence of intra-cristae spaces of different widths indicated that the mitochondria were in a different functional state, as expected in live cells (Figures 6A–C).

In PXE fibroblasts, most of the mitochondria had an elongated morphology, but with few cristae (Figures 6D,E). The presence of round, transversally sectioned mitochondria (Figure 6E) suggests that these organelles have a different 3D organization compared to those in control cells. Moreover, a number of mitochondria exhibit membranes organized in multiple layers, forming separate compartments or concentric “onion-shaped” structures (Figures 6F–I). Altered mitochondrial morphology is known to have consequences on an organelle’s function (i.e., oxidative phosphorylation, Krebs cycle, fatty acid beta-oxidation, heme synthesis, ROS production, ion storage).

It has been demonstrated that, besides the internal structural organization, mitochondria dynamics is also fundamental to cellular functionality, and alterations can be associated with diseases (Duchen, 2004; Chan, 2006; Galloway and Yoon, 2013; Gao et al., 2017) and/or with stress conditions (Blackstone and Chang, 2011; Shutt and McBride, 2013).

**FIGURE 7:**
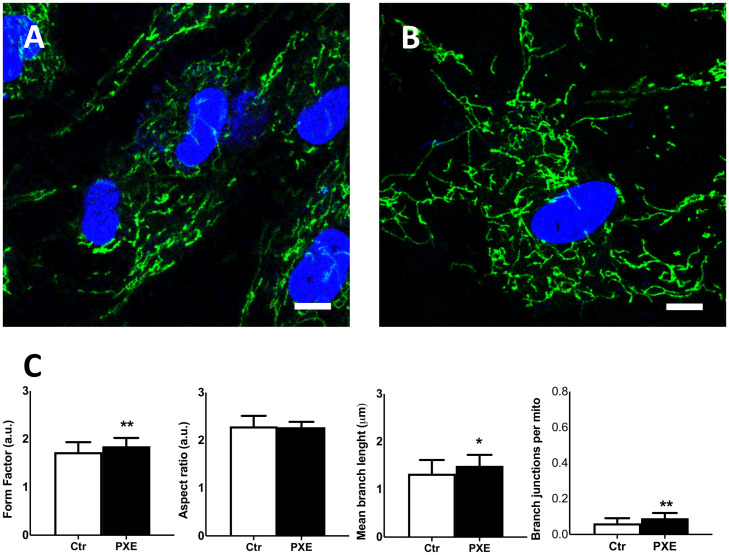
Representative confocal images of mitochondrial topology in control (Ctr) **(A)** and in *Pseudoxanthoma elasticum* (PXE) **(B)**. MitoView Green and Hoechst 33342 were added into the culture medium to stain the mitochondria and the nuclei, respectively. Live-cell images were recorded by confocal microscopy. PXE cells show a more intricate mitochondrial network than control cells. Bar = 10 μm. **(C)** Quantitative analysis and comparison of mitochondrial network connectivity were performed on Ctr and PXE fibroblasts. Data are represented as mean ± SEM of three independent experiments conducted in triplicate on each cell line. *p < 0.05; **p < 0.01 PXE *vs* Ctr.

Therefore, to further investigate mitochondria morphology and topology, fibroblasts were stained with MitoView Green dye (Figure 7). The pathologic cells exhibited a more extended and interconnected mitochondrial network compared to the control cells (Figures 7A,B). Quantitative morphological analyses revealed that, in PXE fibroblasts, aspect ratio did not exhibit significant changes (*p* > 0.05) (Figure 7C), but there was a marked increase in mitochondrial branching (*p* < 0.01) with higher values of branch length (*p* < 0.05) and branch junction (*p* < 0.01) (Figure 7C). CCCP treatment, used as the experimental control of the staining procedure, determined, as expected, a shortening of mitochondria and reduction of mitochondrial branching in both cell lines due to the shift toward fission events as a result of uncoupled oxidative phosphorylation (Friedman and Nunnari, 2014; Supplementary Figure 2).

During cell cycle and cellular lifespan, the mitochondria undergo continuous fission and fusion events leading to different mitochondrial morphologies from small organelles to large and complex networks, respectively (Liu et al., 2020). Mitochondrial fission is essential for growing and dividing cells and serves to eliminate damaged organelles through mitophagy (Twig and Shirihai, 2011), whereas mitochondrial fusion allows the exchange of DNA, proteins, and metabolites; moreover, in aged cells, it can represent an adaptation to impaired mitochondrial biogenesis (Mai et al., 2010), enabling the cells to increase in bioenergetic efficiency to maintain ATP production and to preserve cell viability (Rambold et al., 2011).

The present results indicate an unbalance between mitochondrial fission and fusion in pathologic cells which appear more prone to fusion events, possibly as a consequence of cellular stress response (Tondera et al., 2009; Rambold et al., 2011) due to high intracellular ROS levels and an altered mitochondrial respiratory rate (Yoon et al., 2006). Mitochondrial fusion has been associated with increased glutathione disulfide (GSSG) levels (Rebrin et al., 2003; Gutscher et al., 2008), and it has been demonstrated that, in turn, GSSG and ROS further induce mitochondrial fusion (Koopman et al., 2005; Shutt et al., 2012). Consistently, PXE is characterized by mild chronic oxidative stress due to high ROS content, enhanced protein oxidation and carbamylation, and lipid peroxidation with a shift of the ratio GSSG/GSH toward the oxidized form (Pasquali-Ronchetti et al., 2006; Garcia-Fernandez et al., 2008; Li Q. et al., 2008).

## Conclusion

Although this study was performed on a limited number of cases, the use of integrated technical approaches (i.e., proteomic, biochemical, and morphological analyses) revealed that PXE fibroblasts grown in standard medium were characterized by a peculiar protein signature consistent with altered mitochondrial structure and bioenergenetics. In particular, the PXE mitochondria formed large and branched networks, being more prone to fusion events compared to controls. Fusion is closely linked to mitochondrial Δψ_m_ that, in turn, can regulate calcium transport and accumulation. Consistently, the presence of elongated mitochondria and increased Δψ_m_ were associated with the decreased mitochondrial free calcium observed in PXE compared to controls. Moreover, PXE was characterized by mild oxidative stress, which can modify mitochondrial membrane fluidity and structure and consequently ETC efficiency (i.e., decreased OCR).

It could be argued if changes in PXE fibroblasts and PXE mitochondria are the consequence or the cause of the calcified environment. It cannot be excluded that mineralized tissues exert epigenetic regulatory mechanisms; nevertheless, it has to be pointed out that, in the PXE mouse model, some fibroblast phenotypic alterations are present already before the onset of calcification (Boraldi et al., 2014b) and that *in vitro*-cultured fibroblasts, also from clinically unaffected skin of PXE patients, exhibit a number of features different from healthy control cells (Quaglino et al., 2000).

In PXE, dermal fibroblasts are characterized by changes in cytoskeletal organization (Baccarani Contri et al., 1993) and in cell–matrix interactions (Quaglino et al., 2000). These differences can influence the mitochondria, and it is well known that the mitochondria can modify their functions, depending also on the local qualitative and quantitative characteristics of the extracellular matrix (i.e., glycosaminoglycans, elastin-associated components, proteolytically degraded peptides) (Tiozzo Costa et al., 1988; Gheduzzi et al., 2005) through an interconnected sensory system involving cell–matrix interactions and cytoskeletal components (de Cavanagh et al., 2009). The mitochondria, in fact, show a great heterogeneity depending on the tissue, on the energy requirements, and on the mechanical forces that can be transduced from the environment to the cells up to the mitochondria (Johnson et al., 2007; Feng and Kornmann, 2018).

Maladaptation of mitochondrial response to environmental changes may contribute to pathologic conditions and to the development of a pro-osteogenic context (i.e., activation of bone-related signaling pathways, altered balance between pro- and anti-osteogenic factors, sub-chronic inflammatory stimuli increasing elastin damages/degradation) (de Cavanagh et al., 2009; Lee et al., 2020) or may contribute to the increased susceptibility of fibroblasts to pro-osteogenic signals (Boraldi et al., 2014a), favoring ectopic calcification.

Interestingly, mitochondria are key players in the development of the aging process, and fibroblasts cultured from aged individuals or aged *in vitro* are more prone to calcify (Boraldi et al., 2015). Since PXE can be also regarded as a premature aging syndrome (Garcia-Fernandez et al., 2008; Tiemann et al., 2020), it can be hypothesized that mitochondria represent a common link contributing to the development of ectopic calcification in aging and in diseases.

Within this context, it has to be underlined that PXE as well as aged cells are characterized by mitochondria-generated oxidative stress and protein carbamylation (Boraldi et al., 2009; Carracedo et al., 2018) and that these two conditions have been demonstrated to inhibit the expression of ectonucleotide pyrophosphate/phosphodiesterase 1, a potent calcification inhibitor, thus favoring the mineralization of soft connective tissues (Mori et al., 2018).

Even though several reports have demonstrated that antioxidants can ameliorate ectopic calcification (Chao et al., 2019), actually, a number of attempts aiming to limit the calcification process in PXE failed to be effective (Li Q. et al., 2008), suggesting that results may depend on the specific context. Since Δψ_m_ is controlled by proton pumps and by Δψ_m_ discharge, which regulate the balance between ATP synthesis and hydrolysis, it could be suggested that optimal Δψ_m_ values could be re-established by “mild uncouplers.” This approach could lower Δψ_m_ and reduce ROS accumulation, maintaining adequate amounts of ATP (Zorova et al., 2018) and ameliorating cell metabolism, with a positive effect on t number of signaling pathways associated to pathologic calcification.

## Data Availability Statement

The datasets presented in this study can be found in online repositories. The mass spectrometry proteomics data have been deposited to the ProteomeXchange Consortium *via* the PRIDE partner repository with the dataset identifier PXD021647.

## Ethics Statement

Studies involving human cryo-stored cells were performed in accordance with the principles of the Declaration of Helsinki. All participants at time of sampling provided written informed consent.

## Author Contributions

FDL and FB conceived and designed the experiments. FDL, MG-F and LE performed the experiments. FDL, FB, PV, and DQ analyzed the data. FB, FDL, and DQ wrote the manuscript. All authors listed have made a substantial, direct and intellectual contribution to the work, and approved it for publication.

## Conflict of Interest

The authors declare that the research was conducted in the absence of any commercial or financial relationships that could be construed as a potential conflict of interest. The handling editor declared a past collaboration with several of the authors PV and DQ.

## Footnotes

1 https://github.com/AhsenChaudhry/Mitochondria-Analyzer
