# Supplementary material for: Relationship Between Mitochondrial Structure and Bioenergetics in Pseudoxanthoma elasticum Dermal Fibroblasts
Source: Front Cell Dev Biol. 2020 Dec 17;8:610266. doi: 10.3389/fcell.2020.610266 (PMC7773789; doi:10.3389/fcell.2020.610266)
Supplement: Supplementary file 1 [file Data_Sheet_1.PDF]

Table S1. Protein list identified in mitochondria isolated from control (Ctr) and PXE fibroblasts. Mitochondrial proteins are reported in bold.

Protein name	Gene name	Description	SCORE Ctr_A	SCORE Ctr_B	SCORE Ctr_C	SCORE PXE_A	SCORE PXE_B	SCORE PXE_C	Mass Ctr_A	Mass Ctr_B	Mass Ctr_C	Mass PXE_A	Mass PXE_B	Mass PXE_C	Num. of matches Ctr_A	Num. of matches Ctr_B	Num. of matches Ctr_C	Num. of matches PXE_A	Num. of matches PXE_B	Num. of matches PXE_C	Num. of significant matches Ctr_A	Num. of significant matches Ctr_B	Num. of significant matches Ctr_C	Num. of significant matches PXE_A	Num. of significant matches PXE_B	Num. of significant matches PXE_C	Num. of sequences Ctr_A	Num. of sequences Ctr_B	Num. of sequences Ctr_C	Num. of sequences PXE_A	Num. of sequences PXE_B	Num. of sequences PXE_C	Num. of significant sequences Ctr_A	Num. of significant sequences Ctr_B	Num. of significant sequences Ctr_C	Num. of significant sequences PXE_A	Num. of significant sequences PXE_B	Num. of significant sequences PXE_C
**1433E_HUMAN**	YWHAE	14-3-3 protein epsilon	28	72	58	73	113	79	29326	29326	29326	29326	29326	29326	5	7	5	14	12	12	2	5	3	8	7	7	3	4	3	7	5	4	1	3	2	4	4	4
**1433T_HUMAN**	YWHAQ	14-3-3 protein theta	0	0	0	72	59	61	0	0	0	28032	28032	28032	0	0	0	10	10	11	0	0	0	5	5	5	0	0	0	4	4	4	0	0	0	3	3	3
**1433Z_HUMAN**	YWHAZ	14-3-3 protein zeta/delta	0	61	48	90	58	70	0	27899	27899	27899	27899	27899	0	16	13	16	18	20	0	4	2	6	5	6	0	7	6	5	5	5	0	2	2	3	3	3
1A32_HUMAN	HLA-A	HLA class I histocompatibility antigen, A-32 alpha chain	0	0	59	100	107	102	0	0	41307	41307	41307	41307	0	0	24	36	35	35	0	0	1	2	2	2	0	0	5	6	6	6	0	0	1	1	1	1
**3HIDH_HUMAN**	HIBADH	3-hydroxyisobutyrate dehydrogenase, mitochondrial	73	84	76	364	330	372	35705	35705	35705	35705	35705	35705	17	19	16	34	32	32	5	4	5	18	19	15	6	6	6	15	13	12	3	3	3	10	10	8
4F2_HUMAN	SLC3A2	4F2 cell-surface antigen heavy chain	0	30	50	0	30	0	0	68180	68180	0	68180	0	0	4	8	0	4	0	0	1	3	0	1	0	0	1	3	0	2	0	0	1	2	0	1	0
5NTD_HUMAN	NT5E	5'-nucleotidase	126	145	124	157	191	138	63898	63898	63898	63898	63898	63898	17	15	15	30	26	30	8	9	5	8	12	5	11	8	9	13	13	13	5	5	5	5	8	3
**68MP_HUMAN**	MP68	6.8 kDa mitochondrial proteolipid	0	0	32	54	0	35	0	0	6658	6658	0	6658	0	0	4	7	0	8	0	0	1	2	0	1	0	0	2	2	0	2	0	0	1	1	0	1
AAAT_HUMAN	SLC1A5	Neutral amino acid transporter B(0)	100	55	122	93	75	58	57018	57018	57018	57018	57018	57018	14	12	10	18	15	17	4	2	3	2	2	1	4	4	3	4	2	5	2	1	2	1	1	1
**AATM_HUMAN**	GOT2	Aspartate aminotransferase, mitochondrial	370	345	318	603	579	486	47886	47886	47886	47886	47886	47886	33	29	31	73	74	77	12	12	13	30	27	25	14	14	14	22	22	21	6	6	6	11	10	11
**ABHDA_HUMAN**	ABHD10	Mycophenolic acid acyl-glucuronide esterase, mitochondrial	46	0	0	99	43	43	34253	0	0	34253	34253	34253	4	0	0	5	6	4	2	0	0	4	2	4	1	0	0	1	2	1	1	0	0	1	1	1
**ACAD9_HUMAN**	ACAD9	Acyl-CoA dehydrogenase family member 9, mitochondrial	34	0	0	99	82	74	69344	0	0	69344	69344	69344	25	0	0	57	56	50	1	0	0	6	5	3	7	0	0	18	12	12	1	0	0	3	3	2
**ACADM_HUMAN**	ACADM	Medium-chain specific acyl-CoA dehydrogenase, mitochondrial	0	0	0	34	35	0	0	0	0	47015	47015	0	0	0	0	7	4	0	0	0	0	2	1	0	0	0	0	4	2	0	0	0	0	2	1	0
**ACADV_HUMAN**	ACADVL	Very long-chain specific acyl-CoA dehydrogenase, mitochondrial	704	742	502	1037	1002	894	70745	70745	70745	70745	70745	70745	73	72	67	109	108	115	27	30	25	49	45	40	23	23	20	34	30	35	13	14	13	21	19	19
**ACO13_HUMAN**	ACOT13	Acyl-coenzyme A thioesterase 13	0	0	0	66	40	89	0	0	0	15065	15065	15065	0	0	0	6	6	8	0	0	0	2	3	4	0	0	0	4	4	4	0	0	0	2	2	2
**ACON_HUMAN**	ACO2	Aconitate hydratase, mitochondrial	267	350	287	759	626	526	86113	86113	86113	86113	86113	86113	43	48	35	71	72	67	18	21	14	34	34	26	23	24	17	31	32	28	11	10	9	15	15	12
**ACOT2_HUMAN**	ACOT2	Acyl-coenzyme A thioesterase 2, mitochondrial	0	0	30	64	66	0	0	0	53584	53584	53584	0	0	0	5	18	20	0	0	0	1	4	5	0	0	0	3	10	7	0	0	0	1	4	3	0
**ACOT9_HUMAN**	ACOT9	Acyl-coenzyme A thioesterase 9, mitochondrial	69	64	46	237	140	170	50269	50269	50269	50269	50269	50269	8	12	9	20	25	25	2	4	3	10	10	11	6	7	5	11	13	13	2	3	3	7	6	8
**ACPM_HUMAN**	NDUFAB1	Acyl carrier protein, mitochondrial	138	199	123	178	138	104	17577	17577	17577	17577	17577	17577	9	10	9	20	21	20	7	8	6	9	8	7	3	3	3	5	5	5	2	2	2	3	2	3
ACTB_HUMAN	ACTB	Actin, cytoplasmic 1	3334	3398	2804	2624	2495	2176	42052	42052	42052	42052	42052	42052	262	255	243	206	201	207	149	158	132	130	121	114	29	27	27	25	27	27	21	21	20	19	18	20
ACTC_HUMAN	ACTC1	Actin, alpha cardiac muscle 1	1750	1686	1319	1170	1073	966	42334	42334	42334	42334	42334	42334	176	166	166	140	135	137	91	92	76	70	64	61	24	22	23	20	21	22	17	17	15	13	11	14
**ACTN1_HUMAN**	ACTN1	Alpha-actinin-1	0	294	0	430	425	347	0	103563	0	103563	103563	103563	0	38	0	51	57	60	0	16	0	23	23	21	0	18	0	23	22	23	0	9	0	10	9	8
ACTN4_HUMAN	ACTN4	Alpha-actinin-4	451	346	240	497	564	400	105245	105245	105245	105245	105245	105245	37	36	32	67	67	70	19	22	15	28	29	28	16	15	15	27	25	28	8	11	10	12	12	13
**ADT2_HUMAN**	SLC25A5	ADP/ATP translocase 2	654	554	521	754	789	529	33059	33059	33059	33059	33059	33059	90	84	79	111	117	112	45	39	41	59	60	46	17	19	18	21	22	22	10	11	12	14	14	13
**ADT3_HUMAN**	SLC25A6	ADP/ATP translocase 3	870	652	659	963	946	668	33073	33073	33073	33073	33073	33073	112	107	103	142	143	139	53	47	47	69	68	54	19	19	19	23	24	23	11	12	11	15	14	13
**AGK_HUMAN**	AGK	Acylglycerol kinase, mitochondrial	0	0	33	43	77	44	0	0	47564	47564	47564	47564	0	0	8	26	23	24	0	0	1	2	4	3	0	0	2	6	4	4	0	0	1	1	1	1
AHNK_HUMAN	AHNAK	Neuroblast differentiation-associated protein AHNAK	200	196	174	615	666	554	629213	629213	629213	629213	629213	629213	91	89	72	177	193	200	20	28	21	61	56	45	45	41	40	82	85	87	14	17	16	37	35	28
**AIFM1_HUMAN**	AIFM1	Apoptosis-inducing factor 1, mitochondrial	27	0	43	28	45	41	67144	0	67144	67144	67144	67144	7	0	9	24	29	29	2	0	1	1	3	2	5	0	7	14	14	16	2	0	1	1	3	1
**AL1B1_HUMAN**	ALDH1B1	Aldehyde dehydrogenase X, mitochondrial	295	276	217	226	245	244	57626	57626	57626	57626	57626	57626	41	43	38	36	38	40	16	14	9	10	12	12	15	13	14	12	10	13	9	7	4	5	6	6
**AL1L2_HUMAN**	ALDH1L2	Mitochondrial 10-formyltetrahydrofolate dehydrogenase	521	432	424	749	757	638	102365	102365	102365	102365	102365	102365	43	47	46	86	83	85	23	20	21	38	37	32	21	24	21	38	39	37	13	13	13	19	20	18
**AL7A1_HUMAN**	ALDH7A1	Alpha-aminoadipic semialdehyde dehydrogenase	272	296	133	440	301	323	59020	59020	59020	59020	59020	59020	44	48	44	66	64	64	12	12	8	15	19	15	13	15	12	22	20	19	8	8	5	8	10	8
**AL9A1_HUMAN**	ALDH9A1	4-trimethylaminobutyraldehyde dehydrogenase	0	0	0	68	72	61	0	0	0	54679	54679	54679	0	0	0	29	29	26	0	0	0	3	3	3	0	0	0	9	9	7	0	0	0	2	3	2
**ALDH2_HUMAN**	ALDH2	Aldehyde dehydrogenase, mitochondrial	0	0	0	26	61	39	0	0	0	56859	56859	56859	0	0	0	16	14	14	0	0	0	1	4	1	0	0	0	8	6	8	0	0	0	1	4	1
ALDOA_HUMAN	ALDOA	Fructose-bisphosphate aldolase A	344	325	338	218	316	263	39851	39851	39851	39851	39851	39851	36	34	39	27	37	37	13	16	15	11	14	11	16	16	18	14	16	17	6	10	9	5	7	5
AMPN_HUMAN	ANPEP	Aminopeptidase N	666	697	377	783	844	694	109870	109870	109870	109870	109870	109870	44	44	52	69	70	71	30	30	21	32	33	25	23	23	24	31	32	32	17	16	12	17	17	13
ANT3_HUMAN	SERPINC1	Antithrombin-III	216	191	146	142	137	97	53025	53025	53025	53025	53025	53025	59	63	58	54	52	56	15	18	10	9	8	6	11	11	11	11	11	12	4	4	3	3	3	2
**ANXA1_HUMAN**	ANXA1	Annexin A1	29	43	0	35	0	0	38918	38918	0	38918	0	0	14	13	0	16	0	0	2	1	0	1	0	0	5	5	0	5	0	0	1	1	0	1	0	0
**ANXA2_HUMAN**	ANXA2	Annexin A2	2345	2293	2017	2150	2087	1845	38808	38808	38808	38808	38808	38808	162	168	162	189	189	197	95	90	90	92	89	86	37	38	36	36	35	37	22	19	19	20	22	19
ANXA5_HUMAN	ANXA5	Annexin A5	0	0	0	153	129	73	0	0	0	35971	35971	35971	0	0	0	18	11	11	0	0	0	6	8	2	0	0	0	8	5	4	0	0	0	3	4	1
**AP2B1_HUMAN**	AP2B1	AP-2 complex subunit beta	186	149	81	0	0	0	105398	105398	105398	0	0	0	8	7	11	0	0	0	5	3	2	0	0	0	5	4	7	0	0	0	2	2	2	0	0	0
ARC1B_HUMAN	ARPC1B	Actin-related protein 2/3 complex subunit 1B	0	0	0	54	34	47	0	0	0	41722	41722	41722	0	0	0	6	3	3	0	0	0	1	2	1	0	0	0	4	2	2	0	0	0	1	1	1
ARF6_HUMAN	ARF6	ADP-ribosylation factor 6	39	42	30	53	39	0	20183	20183	20183	20183	20183	0	14	14	15	17	14	0	3	2	1	3	2	0	5	6	6	5	4	0	2	2	1	2	1	0
**ARL8B_HUMAN**	ARL8B	ADP-ribosylation factor-like protein 8B	0	45	48	43	76	38	0	21753	21753	21753	21753	21753	0	6	7	7	13	10	0	2	1	1	4	2	0	3	3	3	5	4	0	1	1	1	3	1
ARPC2_HUMAN	ARPC2	Actin-related protein 2/3 complex subunit 2	35	30	28	29	30	0	34426	34426	34426	34426	34426	0	2	2	1	2	1	0	2	1	1	1	1	0	1	1	1	1	1	0	1	1	1	1	1	0
AT1A1_HUMAN	ATP1A1	Sodium/potassium-transporting ATPase subunit alpha-1	0	30	38	36	0	0	0	114135	114135	114135	0	0	0	11	11	8	0	0	0	1	1	2	0	0	0	6	7	6	0	0	0	1	1	2	0	0
AT2B4_HUMAN	ATP2B4	Plasma membrane calcium-transporting ATPase 4	140	106	89	71	87	51	139030	139030	139030	139030	139030	139030	34	29	31	37	32	31	6	6	4	4	6	3	12	11	12	11	11	10	3	4	3	3	4	2
**ATSEL_HUMAN**	ATP5EP2	ATP synthase subunit epsilon-like protein, mitochondrial	0	0	0	0	36	50	0	0	0	0	5860	5860	0	0	0	0	13	12	0	0	0	0	1	1	0	0	0	0	6	5	0	0	0	0	1	1
**ATSF1_HUMAN**	ATP5F1	ATP synthase F(0) complex subunit B1, mitochondrial	101	126	105	126	120	132	28947	28947	28947	28947	28947	28947	37	38	38	40	51	49	6	8	7	10	11	9	14	15	13	13	14	14	4	4	4	5	6	6
**ATSG1_HUMAN**	ATP5MC1	ATP synthase F(0) complex subunit C1, mitochondrial	0	0	0	42	0	58	0	0	0	14439	0	14439	0	0	0	2	0	2	0	0	0	1	0	1	0	0	0	2	0	2	0	0	0	1	0	1
**ATAD1_HUMAN**	ATAD1	ATPase family AAA domain-containing protein 1	0	0	0	47	44	0	0	0	0	41060	41060	0	0	0	0	6	6	0	0	0	0	1	1	0	0	0	0	3	2	0	0	0	0	1	1	0
**ATD3A_HUMAN**	ATAD3A	ATPase family AAA domain-containing protein 3A	92	113	37	221	308	234	71610	71610	71610	71610	71610	71610	8	12	8	33	39	38	3	7	2	14	19	16	7	8	6	18	18	17	3	5	2	8	10	8
**ATP5H_HUMAN**	ATP5H	ATP synthase subunit d, mitochondrial	167	135	101	199	212	161	18537	18537	18537	18537	18537	18537	38	42	39	47	50	58	13	12	10	16	16	13	12	12	11	11	12	13	6	7	5	6	6	6
**ATP5I_HUMAN**	ATP5I	ATP synthase subunit e, mitochondrial	35	0	52	70	46	54	7928	0	7928	7928	7928	7928	6	0	7	10	9	10	2	0	2	5	3	4	3	0	3	5	4	4	1	0	2	3	2	3
**ATP5L_HUMAN**	ATP5L	ATP synthase subunit g, mitochondrial	75	82	71	98	84	124	11421	11421	11421	11421	11421	11421	7	8	7	12	15	15	2	2	1	4	4	5	4	5	4	4	5	5	2	2	1	2	3	3
**ATPA_HUMAN**	ATP5F1A	ATP synthase subunit alpha, mitochondrial	2287	2172	1931	3128	2914	2478	59828	59828	59828	59828	59828	59828	167	162	158	216	219	211	101	94	87	128	128	110	36	37	38	42	42	41	24	25	23	28	26	26
**ATPB_HUMAN**	ATP5F1B	ATP synthase subunit beta, mitochondrial	2473	2374	1979	3053	2848	2570	56525	56525	56525	56525	56525	56525	149	146	153	190	187	187	114	102	97	136	132	127	30	30	31	31	32	32	24	22	23	25	24	25
**ATPD_HUMAN**	ATP5F1D	ATP synthase subunit delta, mitochondrial	105	49	106	47	89	144	17479	17479	17479	17479	17479	17479	5	4	5	4	7	8	3	2	3	1	3	4	3	2	3	2	3	3	2	1	2	1	2	2
**ATPG_HUMAN**	ATP5F1C	ATP synthase subunit gamma, mitochondrial	231	238	194	234	264	201	33032	33032	33032	33032	33032	33032	28	27	26	45	45	50	11	12	12	15	20	13	13	12	14	15	14	15	5	5	6	8	6	6
[illegible]	[illegible]	[illegible]	[illegible]	[illegible]	[illegible]	[illegible]	[illegible]	[illegible]	[illegible]	[illegible]	[illegible]	[illegible]	[illegible]	[illegible]	[illegible]	[illegible]	[illegible]	[illegible]	[illegible]	[illegible]	[illegible]	[illegible]	[illegible]	[illegible]	[illegible]	[illegible]	[illegible]	[illegible]	[illegible]	[illegible]	[illegible]	[illegible]	[illegible]	[illegible]	[illegible]	[illegible]	[illegible]	[illegible]
