# Supplementary material for: Relationship Between Mitochondrial Structure and Bioenergetics in Pseudoxanthoma elasticum Dermal Fibroblasts
Source: Front Cell Dev Biol. 2020 Dec 17;8:610266. doi: 10.3389/fcell.2020.610266 (PMC7773789; doi:10.3389/fcell.2020.610266)
Supplement: Supplementary file 2 [file Data_Sheet_2.PDF]

Table S2. Common mitochondrial proteins between control and PXE.

Accession	Gene	Description	log_2 fold change
1433E_HUMAN	YWHAE	14-3-3 protein epsilon	0,3992
1433Z_HUMAN	YWHAZ	14-3-3 protein zeta/delta	0,4998
3HIDH_HUMAN	HIBADH	3-hydroxyisobutyrate dehydrogenase, mitochondrial	1,4556
AATM_HUMAN	GOT2	Aspartate aminotransferase, mitochondrial	0,6220
ABHDA_HUMAN	ABHD10	Mycophenolic acid acyl-glucuronide esterase, mitochondrial	2,0194
ACAD9_HUMAN	ACAD9	Acyl-CoA dehydrogenase family member 9, mitochondrial	2,5490
ACADV_HUMAN	ACADVL	Very long-chain specific acyl-CoA dehydrogenase, mitochondrial	0,1358
ACON_HUMAN	ACO2	Aconitate hydratase, mitochondrial	0,2918
ACOT2_HUMAN	ACOT2	Acyl-coenzyme A thioesterase 2, mitochondrial	2,1230
ACOT9_HUMAN	ACOT9	Acyl-coenzyme A thioesterase 9, mitochondrial	0,8958
ACPM_HUMAN	NDUFAB1	Acyl carrier protein, mitochondrial	-0,1869
ACTN1_HUMAN	ACTN1	Alpha-actinin-1	0,9252
ADT2_HUMAN	SLC25A5	ADP/ATP translocase 2	0,0743
ADT3_HUMAN	SLC25A6	ADP/ATP translocase 3	0,3108
AGK_HUMAN	AGK	Acylglycerol kinase, mitochondrial	0,7406
AIFM1_HUMAN	AIFM1	Apoptosis-inducing factor 1, mitochondrial	0,0785
AL1B1_HUMAN	ALDH1B1	Aldehyde dehydrogenase X, mitochondrial	-0,9582
AL1L2_HUMAN	ALDH1L2	Mitochondrial 10-formyltetrahydrofolate dehydrogenase	0,0032
AL7A1_HUMAN	ALDH7A1	Alpha-aminoadipic semialdehyde dehydrogenase	-0,0557
ANXA1_HUMAN	ANXA1	Annexin A1	-1,6935
ANXA2_HUMAN	ANXA2	Annexin A2	-0,6953
ARL8B_HUMAN	ARL8B	ADP-ribosylation factor-like protein 8B	0,6249
AT5F1_HUMAN	ATP5F1	ATP synthase F(0) complex subunit B1, mitochondrial	0,0056
ATD3A_HUMAN	ATAD3A	ATPase family AAA domain-containing protein 3A	0,8425
ATP5H_HUMAN	ATP5H	ATP synthase subunit d, mitochondrial	-0,5830
ATP5I_HUMAN	ATP5I	ATP synthase subunit e, mitochondrial	0,9392
ATP5L_HUMAN	ATP5L	ATP synthase subunit g, mitochondrial	0,2125
ATPA_HUMAN	ATP5F1A	ATP synthase subunit alpha, mitochondrial	-0,4672
ATPB_HUMAN	ATP5F1B	ATP synthase subunit beta, mitochondrial	-0,2456
ATPD_HUMAN	ATP5F1D	ATP synthase subunit delta, mitochondrial	-0,6851
ATPG_HUMAN	ATP5F1C	ATP synthase subunit gamma, mitochondrial	-0,2164
ATPK_HUMAN	ATP5J2	ATP synthase subunit f, mitochondrial	-0,4682
ATPO_HUMAN	ATP5O	ATP synthase subunit O, mitochondrial	-0,2847
BASI_HUMAN	BSG	Basigin	-0,6946
BCAT2_HUMAN	BCAT2	Branched-chain-amino-acid aminotransferase, mitochondrial	0,3202
BIP_HUMAN	HSPA5	Endoplasmic reticulum chaperone BiP	1,6083
C1QBP_HUMAN	C1QBP	Complement component 1 Q subcomponent-binding protein, mitochondrial	-0,0172
CALR_HUMAN	CALR	Calreticulin	-0,0896
CALU_HUMAN	CALU	Calumenin	-2,8351
CH10_HUMAN	HSPE1	10 kDa heat shock protein, mitochondrial	-0,0115
CH60_HUMAN	HSPD1	60 kDa heat shock protein, mitochondrial	-0,1595
CISD1_HUMAN	CISD1	CDGSH iron-sulfur domain-containing protein 1	0,3686
CISY_HUMAN	CS	Citrate synthase, mitochondrial	-0,7425
CLIC4_HUMAN	CLIC4	Chloride intracellular channel protein 4	1,3126
COX2_HUMAN	MT-CO2	Cytochrome c oxidase subunit 2	-0,4240
COX41_HUMAN	COX4I1	Cytochrome c oxidase subunit 4 isoform 1, mitochondrial	-0,5765
COX5A_HUMAN	COX5A	Cytochrome c oxidase subunit 5A, mitochondrial	1,5424
COX5B_HUMAN	COX5B	Cytochrome c oxidase subunit 5B, mitochondrial	0,4567
COX6C_HUMAN	COX6C	Cytochrome c oxidase subunit 6C	-0,1269
CPT1A_HUMAN	CPT1A	Carnitine O-palmitoyltransferase 1, liver isoform	1,8121
CX7A2_HUMAN	COX7A2	Cytochrome c oxidase subunit 7A2, mitochondrial	0,3110
CY1_HUMAN	CYC1	Cytochrome c1, heme protein, mitochondrial	-0,0338
CYC_HUMAN	CYCS	Cytochrome c	0,3211
DBLOH_HUMAN	DIABLO	Diablo homolog, mitochondrial	0,6018
DECR_HUMAN	DECR1	2,4-dienoyl-CoA reductase, mitochondrial	0,0166
DHE3_HUMAN	GLUD1	Glutamate dehydrogenase 1, mitochondrial	0,4914
DLDH_HUMAN	DLD	Dihydrolipoyl dehydrogenase, mitochondrial	0,3837
ECH1_HUMAN	ECH1	Delta(3,5)-Delta(2,4)-dienoyl-CoA isomerase, mitochondrial	1,1221
ECHA_HUMAN	HADHA	Trifunctional enzyme subunit alpha, mitochondrial	0,0506
ECHB_HUMAN	HADHB	Trifunctional enzyme subunit beta, mitochondrial	0,1145
ECHM_HUMAN	ECHS1	Enoyl-CoA hydratase, mitochondrial	0,2405
ECI1_HUMAN	ECI1	Enoyl-CoA delta isomerase 1, mitochondrial	-0,0285
EFTU_HUMAN	TUFM	Elongation factor Tu, mitochondrial	0,4246
ENPL_HUMAN	HSP90B1	Endoplasmin	0,3670
ETFA_HUMAN	ETFA	Electron transfer flavoprotein subunit alpha, mitochondrial	0,5393
ETFB_HUMAN	ETFB	Electron transfer flavoprotein subunit beta	0,4195
ETHE1_HUMAN	ETHE1	Persulfide dioxygenase ETHE1, mitochondrial	0,2440
FUMH_HUMAN	FH	Fumarate hydratase, mitochondrial	0,5157
G3P_HUMAN	GAPDH	Glyceraldehyde-3-phosphate dehydrogenase	-0,9799
GBB1_HUMAN	GNB1	Guanine nucleotide-binding protein G(I)/G(S)/G(T) subunit beta-1	-0,7060
GHITM_HUMAN	GHITM	Growth hormone-inducible transmembrane protein	1,0194
GLSK_HUMAN	GLS	Glutaminase kidney isoform, mitochondrial	-0,6213
GLYM_HUMAN	SHMT2	Serine hydroxymethyltransferase, mitochondrial	0,3275
GRP75_HUMAN	HSPA9	Stress-70 protein, mitochondrial	0,3116
GRPE1_HUMAN	GRPEL1	GrpE protein homolog 1, mitochondrial	-0,8209
GSTK1_HUMAN	GSTK1	Glutathione S-transferase kappa 1	0,1935
HCD2_HUMAN	HSD17B10	3-hydroxyacyl-CoA dehydrogenase type-2	0,6213
HCDH_HUMAN	HADH	Hydroxyacyl-coenzyme A dehydrogenase, mitochondrial	0,6774
HIBCH_HUMAN	HIBCH	3-hydroxyisobutyryl-CoA hydrolase, mitochondrial	-1,8351
HSDL2_HUMAN	HSDL2	Hydroxysteroid dehydrogenase-like protein 2	1,9769
IDH3A_HUMAN	IDH3A	Isocitrate dehydrogenase [NAD] subunit alpha, mitochondrial	-0,5421
IDHP_HUMAN	IDH2	Isocitrate dehydrogenase [NADP], mitochondrial	0,0320
IVD_HUMAN	IVD	Isovaleryl-CoA dehydrogenase, mitochondrial	2,9062
KAD2_HUMAN	AK2	Adenylate kinase 2, mitochondrial	0,7124
KAD3_HUMAN	AK3	GTP:AMP phosphotransferase AK3, mitochondrial	-0,0818
KAD4_HUMAN	AK4	Adenylate kinase 4, mitochondrial	1,0194
KPYM_HUMAN	PKM	Pyruvate kinase PKM	2,0427
LETM1_HUMAN	LETM1	Mitochondrial proton/calcium exchanger protein	2,0223
LONM_HUMAN	LONP1	Lon protease homolog, mitochondrial	1,2209
LPPRC_HUMAN	LRPPRC	Leucine-rich PPR motif-containing protein, mitochondrial	0,7837
M2OM_HUMAN	SLC25A11	Mitochondrial 2-oxoglutarate/malate carrier protein	-0,2733
MACD1_HUMAN	MACROD1	O-acetyl-ADP-ribose deacetylase MACROD1	0,7406
MCU_HUMAN	MCU	Calcium uniporter protein, mitochondrial	1,1859
MDHM_HUMAN	MDH2	Malate dehydrogenase, mitochondrial	-0,1766
MGST3_HUMAN	MGST3	Microsomal glutathione S-transferase 3	0,1971
MIC13_HUMAN	MIC13	MICOS complex subunit MIC13	-0,1701
MIC19_HUMAN	CHCHD3	MICOS complex subunit MIC19	-0,6946
MIC60_HUMAN	IMMT	MICOS complex subunit MIC60	0,5388
MPCP_HUMAN	SLC25A3	Phosphate carrier protein, mitochondrial	-0,5262
MPPA_HUMAN	PMPCA	Mitochondrial-processing peptidase subunit alpha	0,5701
MPPB_HUMAN	PMPCB	Mitochondrial-processing peptidase subunit beta	-0,1406
MTCH2_HUMAN	MTCH2	Mitochondrial carrier homolog 2	-0,6719
MYH10_HUMAN	MYH10	Myosin-10	-2,6314
MYH9_HUMAN	MYH9	Myosin-9	-0,6591
MYL6_HUMAN	MYL6	Myosin light polypeptide 6	-0,9260
NB5R3_HUMAN	CYB5R3	NADH-cytochrome b5 reductase 3	0,1254
NDUA1_HUMAN	NDUFA1	NADH dehydrogenase [ubiquinone] 1 alpha subcomplex subunit 1	0,4729
NDUA4_HUMAN	NDUFA4	Cytochrome c oxidase subunit NDUFA4	-0,6946
NDUA5_HUMAN	NDUFA5	NADH dehydrogenase [ubiquinone] 1 alpha subcomplex subunit 5	-0,9760
NDUA6_HUMAN	NDUFA6	NADH dehydrogenase [ubiquinone] 1 alpha subcomplex subunit 6	0,2744
NDUA8_HUMAN	NDUFA8	NADH dehydrogenase [ubiquinone] 1 alpha subcomplex subunit 8	0,4523
NDUA9_HUMAN	NDUFA9	NADH dehydrogenase [ubiquinone] 1 alpha subcomplex subunit 9, mitochondrial	-0,2468
NDUAA_HUMAN	NDUFA10	NADH dehydrogenase [ubiquinone] 1 alpha subcomplex subunit 10, mitochondrial	0,0931
NDUAD_HUMAN	NDUFA13	NADH dehydrogenase [ubiquinone] 1 alpha subcomplex subunit 13	-0,4146
NDUB8_HUMAN	NDUFB8	NADH dehydrogenase [ubiquinone] 1 beta subcomplex subunit 8, mitochondrial	-2,3313
NDUBA_HUMAN	NDUFB10	NADH dehydrogenase [ubiquinone] 1 beta subcomplex subunit 10	2,1126
NDUS1_HUMAN	NDUFS1	NADH-ubiquinone oxidoreductase 75 kDa subunit, mitochondrial	1,3332
NDUS2_HUMAN	NDUFS2	NADH dehydrogenase [ubiquinone] iron-sulfur protein 2, mitochondrial	-0,3110
NDUS3_HUMAN	NDUFS3	NADH dehydrogenase [ubiquinone] iron-sulfur protein 3, mitochondrial	0,8042
NDUS7_HUMAN	NDUFS7	NADH dehydrogenase [ubiquinone] iron-sulfur protein 7, mitochondrial	0,7551
NDUS8_HUMAN	NDUFS8	NADH dehydrogenase [ubiquinone] iron-sulfur protein 8, mitochondrial	1,8561
NDUV1_HUMAN	NDUFV1	NADH dehydrogenase [ubiquinone] flavoprotein 1, mitochondrial	0,2606
NDUV2_HUMAN	NDUFV2	NADH dehydrogenase [ubiquinone] flavoprotein 2, mitochondrial	-1,3454
NIPS1_HUMAN	NIPSNAP1	Protein NipSnap homolog 1	1,2358
NIPS2_HUMAN	NIPSNAP2	Protein NipSnap homolog 2	2,2523
NNTM_HUMAN	NNT	NAD(P) transhydrogenase, mitochondrial	0,3261
NPS3A_HUMAN	NIPSNAP3A	Protein NipSnap homolog 3A	0,9649
NT5D2_HUMAN	NT5DC2	5'-nucleotidase domain-containing protein 2	-1,3454
OAT_HUMAN	OAT	Ornithine aminotransferase, mitochondrial	0,9451
OCAD1_HUMAN	OCIAD1	OCIA domain-containing protein 1	0,3123
ODO1_HUMAN	OGDH	2-oxoglutarate dehydrogenase, mitochondrial	1,1453
ODO2_HUMAN	DLST	Dihydrolipoyllysine-residue succinyltransferase component of 2-oxoglutarate dehydrogenase complex, mitochondrial	1,1440
ODP2_HUMAN	DLAT	Dihydrolipoyllysine-residue acetyltransferase component of pyruvate dehydrogenase complex, mitochondrial	3,0140
ODPA_HUMAN	PDHA1	Pyruvate dehydrogenase E1 component subunit alpha, somatic form, mitochondrial	-0,1914
ODPB_HUMAN	PDHB	Pyruvate dehydrogenase E1 component subunit beta, mitochondrial	0,5996
OPA1_HUMAN	OPA1	Dynamin-like 120 kDa protein, mitochondrial	2,6151
P5CR1_HUMAN	PYCR1	Pyrroline-5-carboxylate reductase 1, mitochondrial	0,3021
P5CS_HUMAN	ALDH18A1	Delta-1-pyrroline-5-carboxylate synthase	0,5860
PCKGM_HUMAN	PCK2	Phosphoenolpyruvate carboxykinase [GTP], mitochondrial	-0,0193
PDIA1_HUMAN	P4HB	Protein disulfide-isomerase	0,6827
PDIA3_HUMAN	PDIA3	Protein disulfide-isomerase A3	-0,7625
PHB_HUMAN	PHB	Prohibitin	0,2383
PHB2_HUMAN	PHB2	Prohibitin-2	0,0934
PPIB_HUMAN	PPIB	Peptidyl-prolyl cis-trans isomerase B	-1,8208
PRDX1_HUMAN	PRDX1	Peroxiredoxin-1	0,3786
PRDX3_HUMAN	PRDX3	Thioredoxin-dependent peroxide reductase, mitochondrial	0,4453
PRDX5_HUMAN	PRDX5	Peroxiredoxin-5, mitochondrial	-0,0754
PREP_HUMAN	PITRM1	Presequence protease, mitochondrial	2,4410
QCR1_HUMAN	UQCRC1	Cytochrome b-c1 complex subunit 1, mitochondrial	0,5990
QCR2_HUMAN	UQCRC2	Cytochrome b-c1 complex subunit 2, mitochondrial	1,3667
QCR6_HUMAN	UQCRH	Cytochrome b-c1 complex subunit 6, mitochondrial	-0,6946
QCR7_HUMAN	UQCRB	Cytochrome b-c1 complex subunit 7	-0,2226
QCR9_HUMAN	UQCR10	Cytochrome b-c1 complex subunit 9	-0,0285
RAP1B_HUMAN	RAP1B	Ras-related protein Rap-1b	-1,4420
RL10A_HUMAN	RPL10A	60S ribosomal protein L10a	-1,3454
RLA0_HUMAN	RPLP0	60S acidic ribosomal protein P0	-1,9203
RLA2_HUMAN	RPLP2	60S acidic ribosomal protein P2	-2,1799
RM04_HUMAN	MRPL4	39S ribosomal protein L4, mitochondrial	1,0194
RM44_HUMAN	MRPL44	39S ribosomal protein L44, mitochondrial	-0,6946
RMD1_HUMAN	RMDN1	Regulator of microtubule dynamics protein 1	-0,1267
RMD3_HUMAN	RMDN3	Regulator of microtubule dynamics protein 3	0,4597
RS16_HUMAN	RPS16	40S ribosomal protein S16	-2,0494
RS18_HUMAN	RPS18	40S ribosomal protein S18	-2,3596
SAM50_HUMAN	SAMM50	Sorting and assembly machinery component 50 homolog	1,1394
SCMC1_HUMAN	SLC25A24	Calcium-binding mitochondrial carrier protein SCaMC-1	-0,4627
SCOT1_HUMAN	OXCT1	Succinyl-CoA:3-ketoacid coenzyme A transferase 1, mitochondrial	0,8364
SCPDL_HUMAN	SCCPDH	Saccharopine dehydrogenase-like oxidoreductase	0,8127
SCRB2_HUMAN	SCARB2	Lysosome membrane protein 2	0,6426
SDHA_HUMAN	SDHA	Succinate dehydrogenase [ubiquinone] flavoprotein subunit, mitochondrial	0,9868
SDHB_HUMAN	SDHB	Succinate dehydrogenase [ubiquinone] iron-sulfur subunit, mitochondrial	1,7702
SFXN1_HUMAN	SFXN1	Sideroflexin-1	2,4399
SFXN3_HUMAN	SFXN3	Sideroflexin-3	1,6806
SODM_HUMAN	SOD2	Superoxide dismutase [Mn], mitochondrial	1,9865
SPRY4_HUMAN	SPRYD4	SPRY domain-containing protein 4	-0,7775
SSBP_HUMAN	SSBP1	Single-stranded DNA-binding protein, mitochondrial	0,0913
STML2_HUMAN	STOML2	Stomatin-like protein 2, mitochondrial	1,0192
SUCA_HUMAN	SUCLG1	Succinate--CoA ligase [ADP/GDP-forming] subunit alpha, mitochondrial	0,9995
SUCB1_HUMAN	SUCLA2	Succinate--CoA ligase [ADP-forming] subunit beta, mitochondrial	1,4887
SUCB2_HUMAN	SUCLG2	Succinate--CoA ligase [GDP-forming] subunit beta, mitochondrial	1,0381
SYIM_HUMAN	IARS2	Isoleucine--tRNA ligase, mitochondrial	1,4536
TBB5_HUMAN	TUBB	Tubulin beta chain	0,2072
THIL_HUMAN	ACAT1	Acetyl-CoA acetyltransferase, mitochondrial	1,1265
THIM_HUMAN	ACAA2	3-ketoacyl-CoA thiolase, mitochondrial	0,7002
THIOM_HUMAN	TXN2	Thioredoxin, mitochondrial	0,7164
TIM44_HUMAN	TIMM44	Mitochondrial import inner membrane translocase subunit TIM44	2,1653
TIM50_HUMAN	TIMM50	Mitochondrial import inner membrane translocase subunit TIM50	0,8054
TM14C_HUMAN	TMEM14C	Transmembrane protein 14C	-0,5359
TOM22_HUMAN	TOMM22	Mitochondrial import receptor subunit TOM22 homolog	0,2851
TOM40_HUMAN	TOMM40	Mitochondrial import receptor subunit TOM40 homolog	0,7203
TOM70_HUMAN	TOMM70	Mitochondrial import receptor subunit TOM70	-0,6946
TPP1_HUMAN	TPP1	Tripeptidyl-peptidase 1	0,3394
TRAP1_HUMAN	TRAP1	Heat shock protein 75 kDa, mitochondrial	1,0736
TXTP_HUMAN	SLC25A1	Tricarboxylate transport protein, mitochondrial	0,2124
UCRI_HUMAN	UQCRFS1	Cytochrome b-c1 complex subunit Rieske, mitochondrial	0,5336
VDAC1_HUMAN	VDAC1	Voltage-dependent anion-selective channel protein 1	0,0503
VDAC2_HUMAN	VDAC2	Voltage-dependent anion-selective channel protein 2	0,2058
VDAC3_HUMAN	VDAC3	Voltage-dependent anion-selective channel protein 3	0,3264
VIME_HUMAN	VIM	Vimentin	-1,3556
