# Supplementary material for: Relationship Between Mitochondrial Structure and Bioenergetics in Pseudoxanthoma elasticum Dermal Fibroblasts
Source: Front Cell Dev Biol. 2020 Dec 17;8:610266. doi: 10.3389/fcell.2020.610266 (PMC7773789; doi:10.3389/fcell.2020.610266)
Supplement: Supplementary file 3 [file Data_Sheet_3.PDF]

Table S3. Unique mitochondrial proteins found in control (Ctr) or in PXE.

Accession	Gene	Description
**Unique proteins in Ctr**		
**Accession**	**Gene**	**Description**
AP2B1_HUMAN	AP2B1	AP-2 complex subunit beta
CLH1_HUMAN	CLTC	Clathrin heavy chain 1
EF2_HUMAN	EEF2	Elongation factor 2
RACK1_HUMAN	RACK1	Receptor of activated protein C kinase 1
RL18_HUMAN	RPL18	60S ribosomal protein L18
RL4_HUMAN	RPL4	60S ribosomal protein L4
RL5_HUMAN	RPL5	60S ribosomal protein L5
RL6_HUMAN	RPL6	60S ribosomal protein L6
RS10_HUMAN	RPS10	40S ribosomal protein S10
RS13_HUMAN	RPS13	40S ribosomal protein S13
RS14_HUMAN	RPS14	40S ribosomal protein S14
RS15A_HUMAN	RPS15A	40S ribosomal protein S15a
RS17_HUMAN	RPS17	40S ribosomal protein S17
RS3_HUMAN	RPS3	40S ribosomal protein S3
RS8_HUMAN	RPS8	40S ribosomal protein S8
**Unique proteins in PXE**		
**Accession**	**Gene**	**Description**
1433T_HUMAN	YWHAQ	14-3-3 protein theta
ACADM_HUMAN	ACADM	Medium-chain specific acyl-CoA dehydrogenase, mitochondrial
ACO13_HUMAN	ACOT13	Acyl-coenzyme A thioesterase 13
AL9A1_HUMAN	ALDH9A1	4-trimethylaminobutyraldehyde dehydrogenase
ALDH2_HUMAN	ALDH2	Aldehyde dehydrogenase, mitochondrial
AT5EL_HUMAN	ATP5EP2	ATP synthase subunit epsilon-like protein, mitochondrial
AT5G1_HUMAN	ATP5MC1	ATP synthase F(0) complex subunit C1, mitochondrial
ATAD1_HUMAN	ATAD1	ATPase family AAA domain-containing protein 1
BPHL_HUMAN	BPHL	Valacyclovir hydrolase
C1TM_HUMAN	MTHFD1L	Monofunctional C1-tetrahydrofolate synthase, mitochondrial
CMC1_HUMAN	SLC25A12	Calcium-binding mitochondrial carrier protein Aralar1
CYB5B_HUMAN	CYB5B	Cytochrome b5 type B
DUT_HUMAN	DUT	Deoxyuridine 5'-triphosphate nucleotidohydrolase, mitochondrial
ECI2_HUMAN	ECI2	Enoyl-CoA delta isomerase 2, mitochondrial
ENOA_HUMAN	ENO1	Alpha-enolase
FAHD1_HUMAN	FAHD1	Acylpyruvase FAHD1, mitochondrial
FUND2_HUMAN	FUNDC2	FUN14 domain-containing protein 2
GANAB_HUMAN	GANAB	Neutral alpha-glucosidase AB
GCSH_HUMAN	GCSH	Glycine cleavage system H protein, mitochondrial
GPDM_HUMAN	GPD2	Glycerol-3-phosphate dehydrogenase, mitochondrial
HEM6_HUMAN	CPOX	Oxygen-dependent coproporphyrinogen-III oxidase, mitochondrial
HIG1A_HUMAN	HIGD1A	HIG1 domain family member 1A, mitochondrial
HXK1_HUMAN	HK1	Hexokinase-1
IDH3B_HUMAN	IDH3B	Isocitrate dehydrogenase [NAD] subunit beta, mitochondrial
IDH3G_HUMAN	IDH3G	Isocitrate dehydrogenase [NAD] subunit gamma, mitochondrial
IPYR2_HUMAN	PPA2	Inorganic pyrophosphatase 2, mitochondrial
LACTB_HUMAN	LACTB	Serine beta-lactamase-like protein LACTB, mitochondrial
MECR_HUMAN	MECR	Enoyl-[acyl-carrier-protein] reductase, mitochondrial
MMSA_HUMAN	ALDH6A1	Methylmalonate-semialdehyde dehydrogenase [acylating], mitochondrial
MTCH1_HUMAN	MTCH1	Mitochondrial carrier homolog 1
NB5R1_HUMAN	CYB5R1	NADH-cytochrome b5 reductase 1
NDUAC_HUMAN	NDUFA12	NADH dehydrogenase [ubiquinone] 1 alpha subcomplex subunit 12
NDUB5_HUMAN	NDUFB5	NADH dehydrogenase [ubiquinone] 1 beta subcomplex subunit 5, mitochondrial
NDUF2_HUMAN	NDUFAF2	NADH dehydrogenase [ubiquinone] 1 alpha subcomplex assembly factor 2
NDUS5_HUMAN	NDUFS5	NADH dehydrogenase [ubiquinone] iron-sulfur protein 5
ODBB_HUMAN	BCKDHB	2-oxoisovalerate dehydrogenase subunit beta, mitochondrial
P5CR2_HUMAN	PYCR2	Pyrroline-5-carboxylate reductase 2
PDP1_HUMAN	PDP1	[Pyruvate dehydrogenase [acetyl-transferring]]-phosphatase 1, mitochondrial
PPIF_HUMAN	PPIF	Peptidyl-prolyl cis-trans isomerase F, mitochondrial
PTCD3_HUMAN	PTCD3	Pentatricopeptide repeat domain-containing protein 3, mitochondrial
QCR8_HUMAN	UQCRQ	Cytochrome b-c1 complex subunit 8
QOR_HUMAN	CRYZ	Quinone oxidoreductase
RAB7A_HUMAN	RAB7A	Ras-related protein Rab-7a
RM12_HUMAN	MRPL12	39S ribosomal protein L12, mitochondrial
RM21_HUMAN	MRPL21	39S ribosomal protein L21, mitochondrial
RM39_HUMAN	MRPL39	39S ribosomal protein L39, mitochondrial
RM47_HUMAN	MRPL47	39S ribosomal protein L47, mitochondrial
RM53_HUMAN	MRPL53	39S ribosomal protein L53, mitochondrial
RT10_HUMAN	MRPS10	28S ribosomal protein S10, mitochondrial
RT21_HUMAN	MRPS21	28S ribosomal protein S21, mitochondrial
SAP_HUMAN	PSAP	Prosaposin
SLIRP_HUMAN	SLIRP	SRA stem-loop-interacting RNA-binding protein, mitochondrial
SQOR_HUMAN	SQOR	Sulfide:quinone oxidoreductase, mitochondrial
SYRM_HUMAN	RARS2	Probable arginine--tRNA ligase, mitochondrial
TBA1A_HUMAN	TUBA1A	Tubulin alpha-1A chain
THTR_HUMAN	TST	Thiosulfate sulfurtransferase
TIM16_HUMAN	PAM16	Mitochondrial import inner membrane translocase subunit TIM16
