# Supplementary figures and images for: Relationship Between Mitochondrial Structure and Bioenergetics in Pseudoxanthoma elasticum Dermal Fibroblasts
Source: Front Cell Dev Biol. 2020 Dec 17;8:610266. doi: 10.3389/fcell.2020.610266 (PMC7773789; doi:10.3389/fcell.2020.610266)

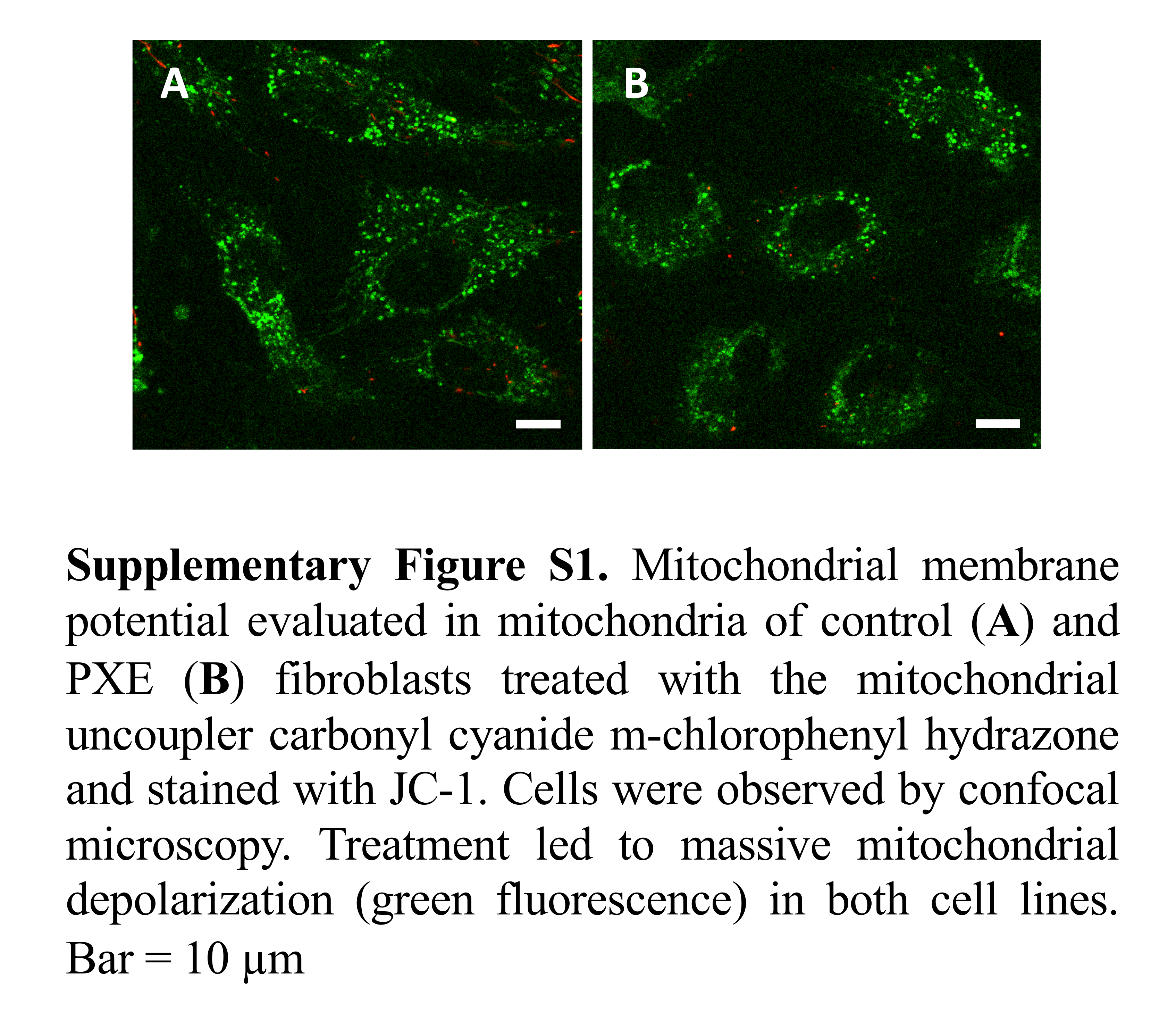

Supplement: Supplementary file 4 [file Image_1.TIF]

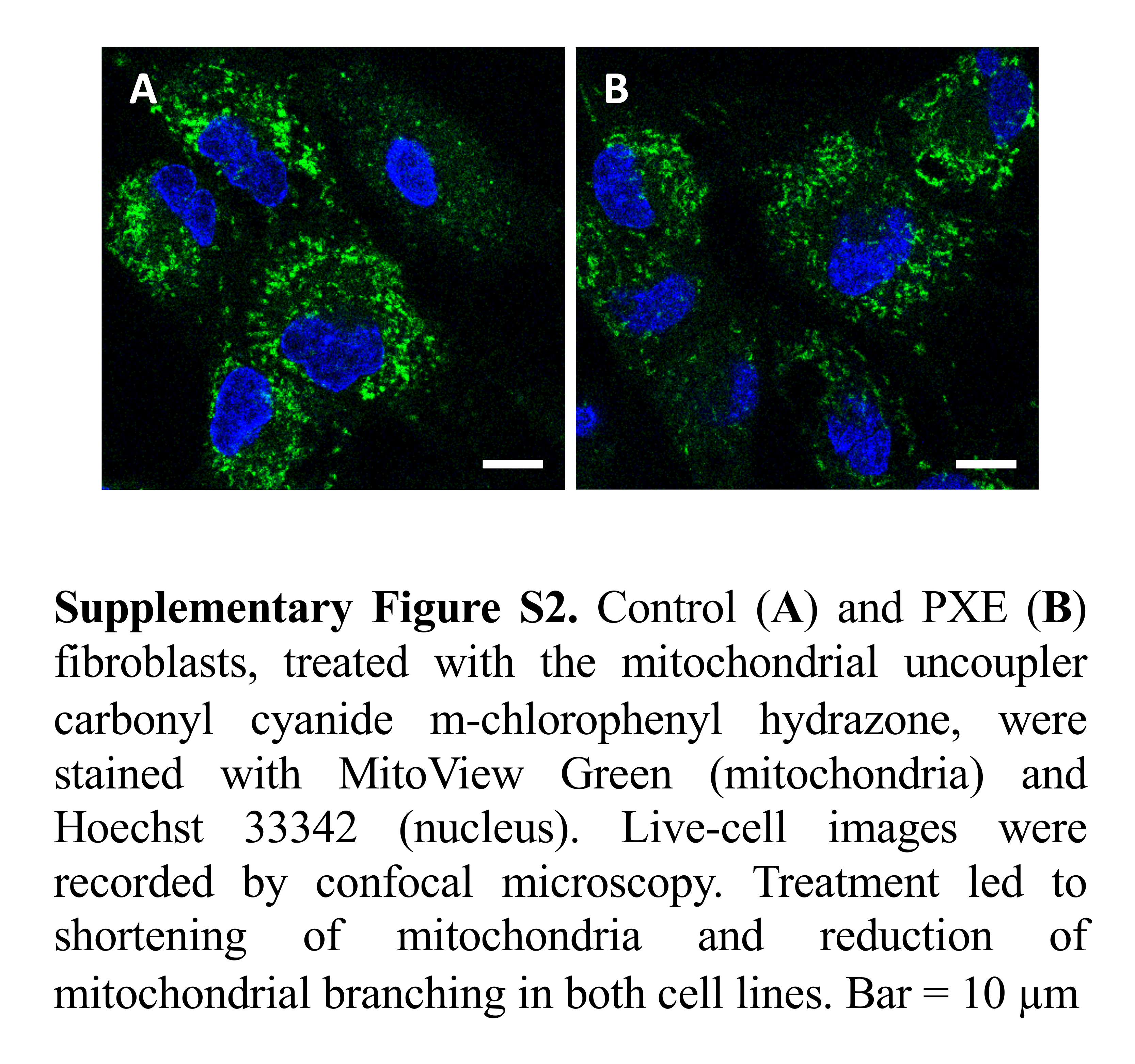

Supplement: Supplementary file 5 [file Image_2.TIF]
